# Onset and window of SARS-CoV-2 infectiousness and temporal correlation with symptom onset: a prospective, longitudinal, community cohort study

**DOI:** 10.1016/S2213-2600(22)00226-0

**Published:** 2022-11

**Authors:** Seran Hakki, Jie Zhou, Jakob Jonnerby, Anika Singanayagam, Jack L Barnett, Kieran J Madon, Aleksandra Koycheva, Christine Kelly, Hamish Houston, Sean Nevin, Joe Fenn, Rhia Kundu, Michael A Crone, Timesh D Pillay, Shazaad Ahmad, Nieves Derqui-Fernandez, Emily Conibear, Paul S Freemont, Graham P Taylor, Neil Ferguson, Maria Zambon, Wendy S Barclay, Jake Dunning, Ajit Lalvani, ATACCC study investigators, Anjna Badhan, Robert Varro, Constanta Luca, Valerie Quinn, Jessica Cutajar, Niamh Nichols, Jessica Russell, Holly Grey, Anjeli Ketkar, Giulia Miserocchi, Chitra Tejpal, Harriet Catchpole, Koji Nixon, Berenice Di Biase, Tamara Hopewell, Janakan Sam Narean, Jada Samuel, Kristel Timcang, Eimear McDermott, Samuel Bremang, Sarah Hammett, Samuel Evetts, Alexandra Kondratiuk

**Affiliations:** a NIHR Health Protection Research Unit in Respiratory Infections, National Heart and Lung Institute, Imperial College London, London, UK; b Section of Virology, Department of Infectious Disease, Imperial College London, London, UK; c Section of Structural and Synthetic Biology, Department of Infectious Disease, Imperial College London, London, UK; d UK Dementia Research Institute Centre for Care Research and Technology, Imperial College London, London, UK; e NIHR Health Protection Research Unit in Modelling and Health Economics, MRC Centre for Global Infectious Disease Analysis, Jameel Institute, Imperial College London, London, UK; f London Biofoundry, Imperial College Translation and Innovation Hub, London, UK; g National Infection Service, UK Health Security Agency, London, UK; h Centre for Experimental Pathogen HostResearch, UCD, Dublin, Ireland; i Department of Virology, Manchester Medical Microbiology Partnership, Manchester Foundation Trust, Manchester Academic Health Sciences Centre, Manchester, UK; j NIHR Health Protection Research Unit in Emerging and Zoonotic Infections, University of Oxford, Oxford, UK

## Abstract

**Background:**

Knowledge of the window of SARS-CoV-2 infectiousness is crucial in developing policies to curb transmission. Mathematical modelling based on scarce empirical evidence and key assumptions has driven isolation and testing policy, but real-world data are needed. We aimed to characterise infectiousness across the full course of infection in a real-world community setting.

**Methods:**

The Assessment of Transmission and Contagiousness of COVID-19 in Contacts (ATACCC) study was a UK prospective, longitudinal, community cohort of contacts of newly diagnosed, PCR-confirmed SARS-CoV-2 index cases. Household and non-household exposed contacts aged 5 years or older were eligible for recruitment if they could provide informed consent and agree to self-swabbing of the upper respiratory tract. The primary objective was to define the window of SARS-CoV-2 infectiousness and its temporal correlation with symptom onset. We quantified viral RNA load by RT-PCR and infectious viral shedding by enumerating cultivable virus daily across the course of infection. Participants completed a daily diary to track the emergence of symptoms. Outcomes were assessed with empirical data and a phenomenological Bayesian hierarchical model.

**Findings:**

Between Sept 13, 2020, and March 31, 2021, we enrolled 393 contacts from 327 households (the SARS-CoV-2 pre-alpha and alpha variant waves); and between May 24, 2021, and Oct 28, 2021, we enrolled 345 contacts from 215 households (the delta variant wave). 173 of these 738 contacts were PCR positive for more than one timepoint, 57 of which were at the start of infection and comprised the final study population. The onset and end of infectious viral shedding were captured in 42 cases and the median duration of infectiousness was 5 (IQR 3–7) days. Although 24 (63%) of 38 cases had PCR-detectable virus before symptom onset, only seven (20%) of 35 shed infectious virus presymptomatically. Symptom onset was a median of 3 days before both peak viral RNA and peak infectious viral load (viral RNA IQR 3–5 days, n=38; plaque-forming units IQR 3–6 days, n=35). Notably, 22 (65%) of 34 cases and eight (24%) of 34 cases continued to shed infectious virus 5 days and 7 days post-symptom onset, respectively (survival probabilities 67% and 35%). Correlation of lateral flow device (LFD) results with infectious viral shedding was poor during the viral growth phase (sensitivity 67% [95% CI 59–75]), but high during the decline phase (92% [86–96]). Infectious virus kinetic modelling suggested that the initial rate of viral replication determines the course of infection and infectiousness.

**Interpretation:**

Less than a quarter of COVID-19 cases shed infectious virus before symptom onset; under a crude 5-day self-isolation period from symptom onset, two-thirds of cases released into the community would still be infectious, but with reduced infectious viral shedding. Our findings support a role for LFDs to safely accelerate deisolation but not for early diagnosis, unless used daily. These high-resolution, community-based data provide evidence to inform infection control guidance.

**Funding:**

National Institute for Health and Care Research.

## Introduction

Widespread community transmission of SARS-CoV-2 continues to occur, even in populations with high levels of immunity. Reducing transmission remains central to the public health response; however, as virus circulation becomes endemic, there is a need for a pragmatic approach, ideally limiting self-isolation to the duration of infectiousness. Delineation of the window of infectiousness and how the degree of infectiousness changes with time since infection, symptom onset, and diagnostic test results is therefore fundamental to developing a better understanding of transmission and more effective, evidence-based infection control policies.

Mathematical modelling based on scarce empirical evidence and key assumptions has substantially driven isolation and testing policy internationally.^1^, ^2^ Cross-sectional data have been extrapolated to impute infectiousness from RT-PCR viral load and antigen-detecting lateral flow device (LFD) results, with the assumption that these relationships stay the same throughout the course of infection.^3^ Very few studies have longitudinally assessed the presence of infectious SARS-CoV-2 through the course of infection, and none have serially quantified infectious virus in mild community cases, which account for most transmission globally.^4^, ^5^

**Research in context**

**Evidence before this study**

A search of PubMed from database inception to March 10, 2022, for studies with the keywords “individuals” or “persons”, “viral dynamics”, and “SARS-CoV-2” in the title or abstract, without language or other restrictions, identified 19 results (with no duplicates). All 19 results were evaluated, with ten deemed to be relevant on the basis that they were longitudinal human studies of COVID-19. Of these, six studies analysed the viral RNA trajectories in participants who were PCR positive from study onset (ie, prevalent cases), thereby missing the crucial growth phase of viral shedding. Three studies reported viral RNA trajectories in a cohort of professional US sports players, including cases who became PCR positive during the study period, but without measuring cultivable infectious virus. Only one cohort study, involving prospective surveillance of college-enrolled students, did daily RT-PCR in addition to measuring infectious virus by in vitro culture.A second search, from inception to March 10, 2022, using title or abstract terms “viral kinetics”, and “SARS-CoV-2”, without language or other restrictions, returned 46 results (with one duplicate result), 17 of which were deemed to be relevant. Of these, 16 focused on viral RNA trajectories in prevalent cases. One study captured the onset of viral shedding with serial saliva and nasal sampling in a small cohort of seven cases, but did not attempt to measure culturable infectious virus.A third search, from inception to March 10, 2022, using title or abstract terms “antigen” or “lateral flow”, “SARS-CoV-2”, and “infectiousness”, without language or other restrictions, returned 25 results (with no duplicates). 11 were deemed to be relevant on the basis that they attempted to link lateral flow device (LFD) results with individual infectiousness. Of these, eight made assumptions of infectiousness based on viral copy number quantified by RT-PCR. The other three studies measured LFD sensitivity relative to in vitro cell culture (using binary viral culture success or failure rather than quantitative measures of infectiousness) and found higher sensitivity than when compared with RT-PCR, but crucially only evaluated LFDs cross-sectionally rather than across the course of infection.Finally, we reviewed the human challenge study in which SARS-CoV-2-naive healthy volunteers were inoculated with a standardised dose of a specific SARS-CoV-2 strain directly into the nares. This study included both daily RT-PCR and viral culture of serial samples and found that infectious virus could be recovered from participants during a median period of 6·5 days. However, it is unknown whether these observations are generalisable to naturally acquired infection in the real-world setting of community contacts of COVID-19 cases, where most transmission occurs globally with broader demographics.

**Added value of this study**

To our knowledge, this is the first study to serially quantify both viral RNA and infectious, culturable virus from the start of naturally acquired SARS-CoV-2 infection. We did RT-PCR, in vitro cell culture-based quantitative plaque assays to enumerate infectious virus, and lateral flow antigen tests from the upper respiratory tract of 57 recently exposed cases in a real-world community setting, capturing the early viral growth phase during which most transmission occurs. This enabled us to define the window of infectiousness (median duration of infectiousness 5 [IQR 3–7] days) and its temporal relationship to symptom onset, which revealed that two-thirds of cases are still infectious 5 days post-symptom onset and one-third at 7 days. LFDs have poor sensitivity for detecting culturable virus during the growth phase of infection (67% [95% CI 59–75]), but high sensitivity during the decline phase (92% [86–96]), supporting a role for LFDs in de-isolation but not for early diagnosis, unless used daily.

**Implications of all the available evidence**

Our results uniquely define the window and kinetics of SARS-CoV-2 infectiousness in naturally acquired infection. Our findings moreover suggest that the recent observations in the controlled experimental human challenge model are largely generalisable to community COVID-19 cases. However, there was wider inter-individual variability in the duration and amount of infectious viral shedding in our larger, real-world cohort. This probably reflects the greater demographic heterogeneity of the community cases as well as the variability in the infecting dose, and route, for transmission events in the community compared with the highly controlled experimental inoculation of pre-selected healthy volunteers in the challenge model. By delineating the period of infectiousness in mild COVID-19, and its correlation with symptom onset and commonly used diagnostic tests, our findings enable calibration of isolation guidance to the infectious window.

Identifying such cases from the earliest timepoints after exposure and densely sampling them thereafter is essential to delineate the trajectory of infectious viral shedding. However, this is operationally challenging in naturally exposed people. The growth phase and peak of viral replication occur very early post-exposure, meaning studies of symptomatic cases cannot capture this crucial window where a large proportion of transmission occurs.^6^, ^7^, ^8^

We therefore recruited community contacts recently exposed to PCR-confirmed SARS-CoV-2 cases in the UK; contacts self-performed daily upper respiratory tract (URT) swabs through the course of infection. Here, we analyse the contacts in whom we succeeded in capturing the crucial growth phase and peak of viral replication, with the aim of characterising the window of SARS-CoV-2 infectiousness and its temporal correlation with symptom onset. Our dataset provides a valuable opportunity to test the public health implications of shortening self-isolation periods to inform national and organisational case management policies, and how these might be facilitated by point-of-care testing with LFDs.

## Methods

### Study design and participants

The Assessment of Transmission and Contagiousness of COVID-19 in Contacts (ATACCC) study was a UK prospective, longitudinal, community cohort study of community contacts of newly diagnosed, PCR-confirmed SARS-CoV-2 index cases, as previously described and detailed (appendix p 11).^9^ ATACCC enrolment spanned two separate time periods: ATACCC1 enrolled contacts from Sept 13, 2020, to March 31, 2021, during the SARS-CoV-2 pre-alpha and alpha variant waves; and ATACCC2 enrolled contacts from May 24, 2021, to Oct 28, 2021, during the delta variant wave.

PCR-positive contacts are hereafter referred to as cases. Household and non-household exposed contacts aged 5 years or older were eligible for recruitment if they could provide informed consent and agree to self-swabbing of the URT. Unvaccinated cases were defined as those who had not received any COVID-19 vaccination before index case symptom onset, our proxy for exposure. Fully vaccinated cases were defined as those who had received their second COVID-19 vaccination 14 days or more before index case symptom onset; none had received booster vaccinations. Cases who received only one dose of vaccine before index symptom onset were excluded.

The study was approved by the Health Research Authority (Research Ethics Committee reference 20/NW/0231) and samples were obtained with written informed consent. All data were housed within a secure research environment and accessed only by approved researchers (SH, JJ, JLB, KJM, SN, and AL).

### Procedures

All participants had mild-to-moderate ambulatory illness, which did not require hospitalisation. Participants completed a daily symptom diary. We defined symptomatic cases as those experiencing any one of the three canonical COVID-19 symptoms (fever, cough, or a loss or change in smell or taste), or at least two of the following symptoms: muscle aches, headache, appetite loss, or sore throat, as per the criteria defined and validated by Houston and colleagues^10^ in the ATACCC cohort and a related community cohort.

All contacts underwent daily, longitudinal URT sampling for up to 20 days. For each contact, viral RNA load was quantified daily by RT-PCR and the ORF1ab cycle threshold (Ct) values were converted to viral genome copies, as previously described.^9^ Daily nose and throat swabs were placed in 3 mL viral transport medium (VTM) of two brands (Copan Diagnostics, Murrieta, CA, USA; or MANTACC, Guangdong, China). SARS-CoV-2 RT-PCR was done on VTM samples on the same day as sample collection by the UK Health Security Agency (UKHSA). Remaining VTMs were stored at –80°C.

All laboratory assays were done by a scientist (JZ) masked to the variant and vaccination status of the contact from which the samples were derived and their timepoint of infection (appendix p 11). Plaque assays were carried out on 90% (547 of 605) of the PCR-positive samples. Samples that were collected in MANTACC brand VTM were not cultured because the medium proved to be toxic to Vero E6 cells and the remaining samples could not be recovered for assay.

Our study provided a unique opportunity to triangulate the kinetics of SARS-CoV-2 RNA viral loads and infectious viral loads with viral antigen-detecting LFDs. We used the Innova LFD (Innova Medical Group, Pasadena, CA, USA) to evaluate the performance of antigen-detecting LFDs. LFDs were done on PCR-positive samples, as well as 1 day before and 1 day after the positive PCR result. Independent validation experiments were carried out at the UKHSA site in Colindale (London, UK) to identify the amount of thawed VTM that would be most likely to give a result consistent with a concentration of virus in fresh samples,^11^ and LFDs were tested by JZ on thawed VTM simultaneously with the plaque assays (appendix pp 11–12).

PCR-positive samples were submitted for viral whole-genome sequencing to assign lineages, as described in the appendix (p 12).

### Outcomes

The primary objective was to define the window of SARS-CoV-2 infectiousness from the onset of infection and its temporal correlation with symptom onset. The secondary outcome was to longitudinally correlate LFD positivity with infectious viral shedding. We compared the infectious virus kinetics (peak viral load, total amount of virus shed, exponential growth rate, exponential decline rate, growth phase duration, decline phase duration, and the ratio of plaque-forming units [PFU] to RNA copies per mL) between vaccinated and unvaccinated cases as a separate exploratory analysis.

### Statistical analysis and modelling

To estimate values and uncertainties of the kinetic parameters, and account for test accuracy and sensitivity, individual fits of RNA viral load and infectious viral load trajectories were implemented using Bayesian hierarchical modelling, as previously described^9^ and further detailed in the appendix (pp 12–13). We computed posterior probabilities (pp) that the mean distributions of viral kinetic parameters were different for infectious viral shedding and viral RNA shedding, and for unvaccinated and vaccinated individuals. For our model, Bayes factors (BF) can be computed as: pp divided by (1 – pp). pp values greater than 0·75 (corresponding to BF>3) were taken as evidence of at least a moderate difference. The association of age, sex, and body-mass index (BMI) with the measured kinetic parameters were assessed with two-sided *t* tests.

### Role of the funding source

The funder of the study had no role in the study design, data collection, data analysis, data interpretation, the writing of the report and the decision to submit.

## Results

**Figure 1:**
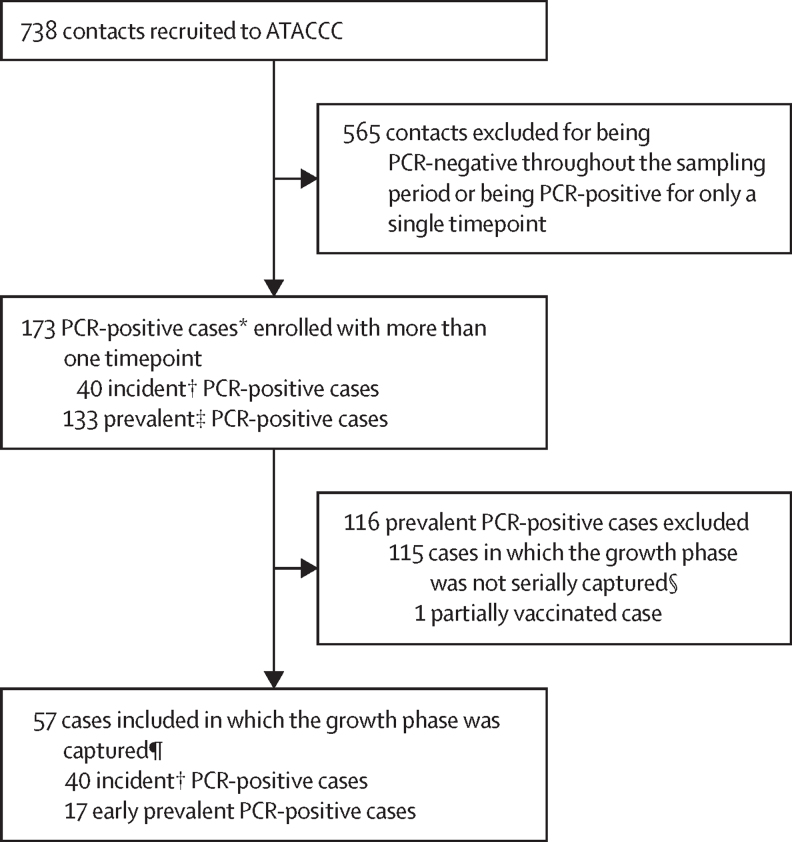
Study profile Flowchart illustrating derivation of the recently infected contacts included in subsequent analyses, from which the growth phase was serially captured. Samples from a total of 57 cases were used. ATACCC=Assessment of Transmission and Contagiousness of COVID-19 in Contacts. *PCR-positive contacts are referred to as cases throughout. †Incident cases were PCR negative on the day of study enrolment and became PCR positive during the study. ‡Prevalent cases were PCR positive from the day of recruitment. §Stringent criteria were applied to the prevalent cases to select only contacts in whom the growth phase was fully captured. ¶13 cases pre-alpha variant, unvaccinated; 12 cases alpha variant, unvaccinated; 7 cases delta variant, unvaccinated; and 25 cases delta variant, fully vaccinated. In some analyses, not all 57 cases were included; see the appendix (p 3) for the full exclusion criteria.

ATACCC1 enrolled 393 contacts from 327 households from Sept 13, 2020, to March 31, 2021, during the SARS-CoV-2 pre-alpha and alpha variant waves; and ATACCC2 enrolled 345 contacts from 215 households from May 24, 2021, to Oct 28, 2021, during the delta variant wave. 738 contacts provided at least one URT swab, of whom 173 (23%) were PCR positive for more than one timepoint, referred to here as cases. 40 (23%) of 173 cases tested PCR negative at enrolment and subsequently became PCR positive, denoted as incident cases. A further 17 cases were PCR positive at enrolment, but met all the following criteria for being captured within the viral growth phase, and are denoted as early prevalent cases: first, low viral load on the day of enrolment (an ORF1ab Ct of >29, corresponding to approximately 72 500 RNA copies per mL); second, an observable increase in viral load after enrolment; and third, PCR positive for three or more timepoints. Thus, in total, 57 cases comprised the final study population (figure 1).

25 (44%) of 57 cases were fully vaccinated and were infected with the delta variant, confirmed by whole-genome sequencing. Of the 32 unvaccinated cases, 13 (41%) were infected with pre-alpha, 12 (38%) with alpha, and seven (22%) with delta SARS-CoV-2 variants, as confirmed by whole-genome sequencing. The final study population was mostly White (51 [89%] of 57), middle-aged (median 41 [IQR 29–49] years), of a healthy BMI (median 25·2 [IQR 21·2–28·8] kg/m^2^), with few reporting comorbidities or pregnancy (13 [23%] of 57; appendix p 2). There were no significant differences in demographic characteristics between the unvaccinated and vaccinated groups.

**Figure 2:**
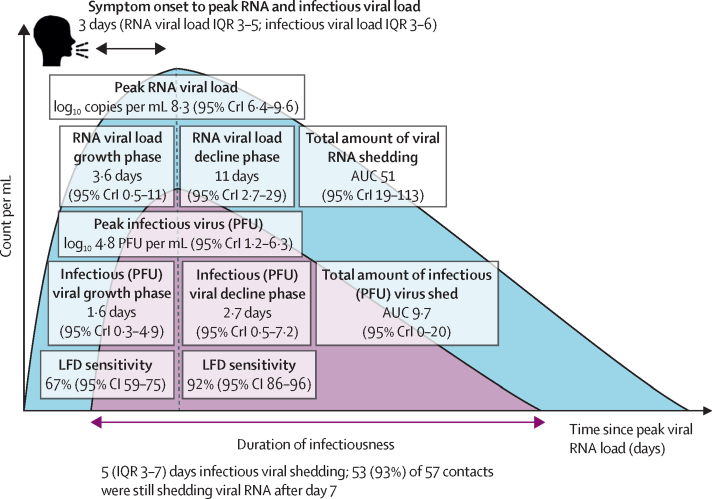
Window and kinetics of SARS-CoV-2 infectiousness in recently infected contacts Graphical summary illustrating the window and kinetics of SARS-CoV-2 infectiousness in recently infected contacts in whom the growth phase was serially captured. The blue curve depicts the typical viral RNA kinetics detected by combined nose and throat swabs, and the purple curve depicts the typical infectious viral kinetics as measured by quantitative plaque assays. All point estimates are medians. The duration of infectiousness (as measured by plaque assay) was measured in 42 cases. Time from symptom onset to peak RNA viral load was measured in 38 cases, and symptom onset to peak infectious viral load in 35 cases. LFD sensitivity was measured against infectious viral shedding during pre-peak to peak viral shedding (n=270 tests) and post-peak viral shedding (n=337 tests). Peak shedding, duration of the growth phase, decline phase, and total shedding were estimated with Bayesian hierarchical modelling (n=57 cases for viral RNA shedding and n=47 cases for infectious viral shedding, as measured by plaque assays). This figure is a simplified summary of the empirical data in figure 3 and the Bayesian modelling data in the appendix p (9). ATACCC=Assessment of Transmission and Contagiousness of COVID-19 in Contacts. AUC=area under the curve. CrI=credible interval. LFD=lateral flow device. PFU=plaque-forming unit.

**Figure 3:**
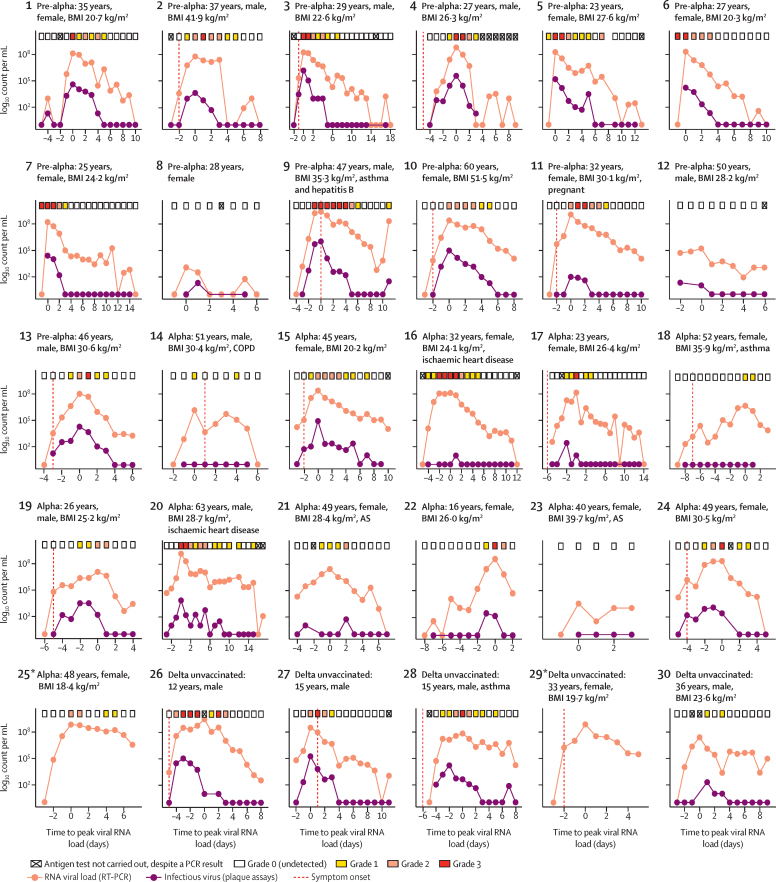

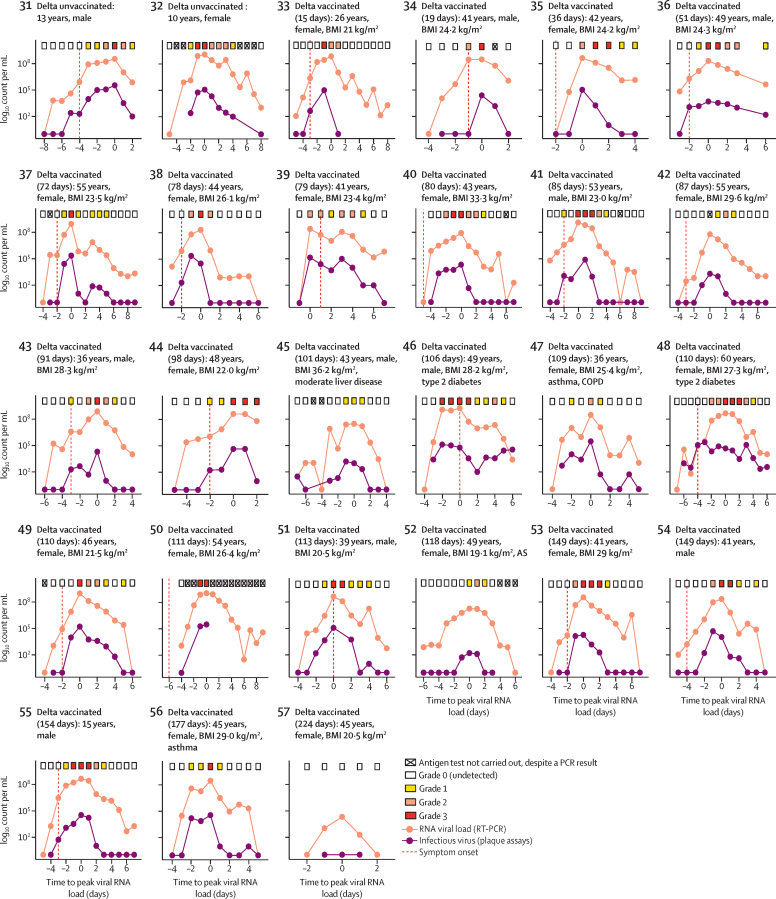
SARS-CoV-2 viral dynamics captured through daily sampling for cases infected with pre-alpha or alpha variants (unvaccinated), or the delta variant (vaccinated and unvaccinated) Samples from a total of 57 cases were used: 13 pre-alpha variant, unvaccinated; 12 alpha variant, unvaccinated; 7 delta variant, unvaccinated; and 25 delta variant, fully vaccinated. All vaccinated cases are denoted as such in the title (n=25) and number of days from receiving the second SARS-CoV-2 vaccine dose to the day of exposure is given in brackets. Age, sex, BMI (calculated for cases aged ≥16 years), and health conditions of the contact, where present, are denoted. Each graph shows the temporal trends for combined nose and throat swab RNA viral load as measured by RT-PCR (orange dots) and infectious virus as measured by plaque assays (purple dots). 34 of 57 cases had information available for the day of symptom onset. For these cases, a red dashed line indicates self-reported symptom onset for at least one of the canonical COVID-19 symptoms (persistent cough, productive cough, loss or change in smell or taste, or fever) or two of the following: sore throat, muscle aches, headache, or appetite loss.^10^ Absence of a dashed line indicates missing symptom-onset information. LFD results on each day are shown in boxes at the top of each graph; red boxes=strong positive, orange boxes=positive, and yellow boxes=weak positive (see appendix p 7 for a detailed visualisation of each grade). AS=asymptomatic. BMI=body-mass index. COPD=chronic obstructive pulmonary disease. LFD=lateral flow device. *Viral transport media from one unvaccinated alpha contact (plot 25) and one vaccinated delta-infected contact (plot 29) could not be cultured due to toxicity of the viral transport medium used for these contacts against Vero E6 cells.

53 (93%) of 57 cases shed viral RNA for over 7 days (figure 2 and figure 3). 51 (93%) of 55 cases had infectious viral shedding detectable as quantifiable PFUs (two cases were not suitable for virus cultivation due to VTM toxicity; figure 3, plots 25 and 29). The onset of infectious viral shedding was captured in 49 (96%) of 51 cases (two cases were culture positive from the day of enrolment, but with a low infectious viral load [<50 PFU per mL; figure 3, plots 12 and 45]). We defined the window of infectiousness as the period in which virus capable of forming PFUs could be detected in the VTM from URT swabs. We were able to characterise the window of infectiousness in 42 (82%) of 51 of cases (appendix p 3) and found that infectious virus was shed for a median of 5 (IQR 3–7) days (figure 2 and figure 3).

There was substantial heterogeneity in the dynamics of infectious viral shedding; however, the median peak RNA viral load (log_10_ 8·4 [IQR 7·9–8·8] RNA copies per mL) and peak infectious viral load (log_10_ 4·5 PFU per mL [3·6–5·1]) attained were fairly homogeneous across cases (appendix p 8). Most cases (32 [63%] of 51) had a peak infectious viral load on the day of peak RNA viral load. Only five (10%) of 51 cases had a peak infectious viral load 1–2 days before peak RNA viral load. All other cases had a peak infectious viral load 1–2 days after the peak RNA viral load.

53 (93%) of 57 cases recorded symptom information and, of these, three (6%; figure 3, plots 21, 23, and 52) were classified as asymptomatic as per the criteria by Houston and colleagues,^10^ one of whom did not shed infectious virus (figure 3, plot 23) and two of whom had peak infectious viral loads below the cohort median. Of the symptomatic cases, 38 (76%) provided a definitive symptom-onset date. Notably, 24 (63%) of 38 cases had PCR-detectable virus before the onset of canonical symptoms, but only nine (25%) of 35 cases (appendix p 3) shed infectious virus before the onset of canonical symptoms. This reduced to seven (20%) of 35 cases that shed infectious virus before the onset of the broader symptom criteria by Houston and colleagues.^10^ Symptom onset was a median of 3 days before both peak RNA viral load and peak infectious viral load (RNA IQR 3–5 days, n=38; PFU IQR 3–6 days, n=35; figure 2 and appendix p 3).

Fits of RNA viral load and infectious viral load trajectories, implemented using Bayesian hierarchical modelling (appendix p 9), are summarised in table 1 and figure 2. We detected no difference in the duration of the growth phase for RNA viral load relative to infectious viral load (PFU 1·6 days, 95% credible interval [CrI] 0·3–4·9; RNA 3·6 days [95% CrI 0·5–11], BF 2·8). The decline phase, however, was longer for RNA viral load than infectious viral load (2·7 days [0·5–7·2]; 11 days [2·7–39·0], 17), and hence accounted for most of the disparity between the overall duration of infectious virus and RNA shedding. We observed a corresponding increase in the decline rate of infectious viral shedding relative to viral RNA shedding, with a BF of 250, but no difference in the growth rates (BF 1). This was confirmed in a sensitivity analysis, in which only participants with at least five positive PFU samples throughout the course of infection were included (growth rate BF 0·42; decline rate BF 330).

**Table 1:** Summary statistics for Bayesian hierarchical modelled viral kinetics for cases, derived from RNA viral load data and plaque assays

	**Viral RNA shedding (n=57)**	**Infectious viral shedding (n=47)**	**Bayes factor**
Peak viral load, log_10_ RNA copies per mL or PFU per mL	8·3 (6·4–9·6)	4.8 (1·2–6·3)	..
Total amount of virus shed, area under the curve of trajectory	51 (19–113)	9·7 (0–20)	..
Exponential growth rate, e-foldings per day	4·9 (1·3–36)	5·0 (1·4–29·8)	1
Exponential decline rate, e-foldings per day	1·6 (0·5–5·3)	2·6 (0·7–10·5)	250
Growth phase duration, days	3·6 (0·5–11)	1·6 (0·3–4·9)	..
Decline phase duration, days	11 (2·7–29)	2·7 (0·5–7·2)	..

Data are posterior mean estimates (95% credible interval). Viral RNA shedding (copies per mL), n=57 (32 unvaccinated and 25 vaccinated); infectious viral shedding (PFU per mL), n=47 (27 unvaccinated and 20 vaccinated). Ten cases were excluded entirely from the infectious viral shedding (PFU) Bayesian hierarchical modelling for the following reasons: toxicity of the viral transport medium used for these contacts against Vero E6 cells (figure 3, plots 25 and 29); not shedding virus capable of forming PFUs (figure 3, plots 14, 18, 23, and 57); and not having enough PFU data during the decline phase for modelling (figure 3, plots 36, 46, 48, and 50). Bayes factors were calculated as: pp divided by (1 – pp), with pp being the posterior probability that the group means of the PFU growth and decline rates were larger than the respective RNA viral load rates. PFU=plaque-forming unit.

Within our overall cohort (n=57), RNA viral load growth rate positively correlated with the peak RNA viral load (correlation coefficient 0·15 [95% CrI –0·16 to 0·43], BF 4·7) and negatively with the RNA viral load decline rates (–0·34 [–0·57 to –0·06], 95; appendix p 4), where the BFs were calculated using the pp that the correlation coefficients were different from zero. Growth rate of infectious viral loads (n=47) also positively correlated with peak infectious viral loads (correlation coefficient 0·36 [95% CrI –0·06 to 0·66], BF 20) and negatively with infectious viral load decline rate (–0·46 [–0·71 to –0·15], 260; appendix p 5).

We hypothesised that the viability of virions might attenuate over the course of infection due to local mucosal immune responses. The ratio of RNA copies per mL to detectable PFU per mL changed during infection (appendix pp 3, 10), with the log RNA copies per mL to PFU per mL ratio significantly increasing with time since the first positive PCR result (n=49 cases, regression gradient coefficient 1·65; p<0·0001). Thus, nearly 100 times more RNA copies are required to generate a single plaque-forming virus 10 days after first PCR positivity. This was true in both unvaccinated and vaccinated cases (p=0·77, analysis of covariance test).

Age did not significantly associate, and sex and BMI only weakly associated, with the measured kinetic parameters with two-sided *t* tests (appendix p 5). We had limited power to detect differences by vaccination status. Although we did not observe any differences in the peak, growth phase, or decline phase of infectious viral load or RNA viral load between vaccinated and unvaccinated cases, the decline rate of infectious viral load was somewhat faster in vaccinated cases (vaccinated 2·1 [95% CrI 0·6–5·0]; unvaccinated 4·7 [1·3–13], BF 4·1; appendix p 6).

**Figure 4:**
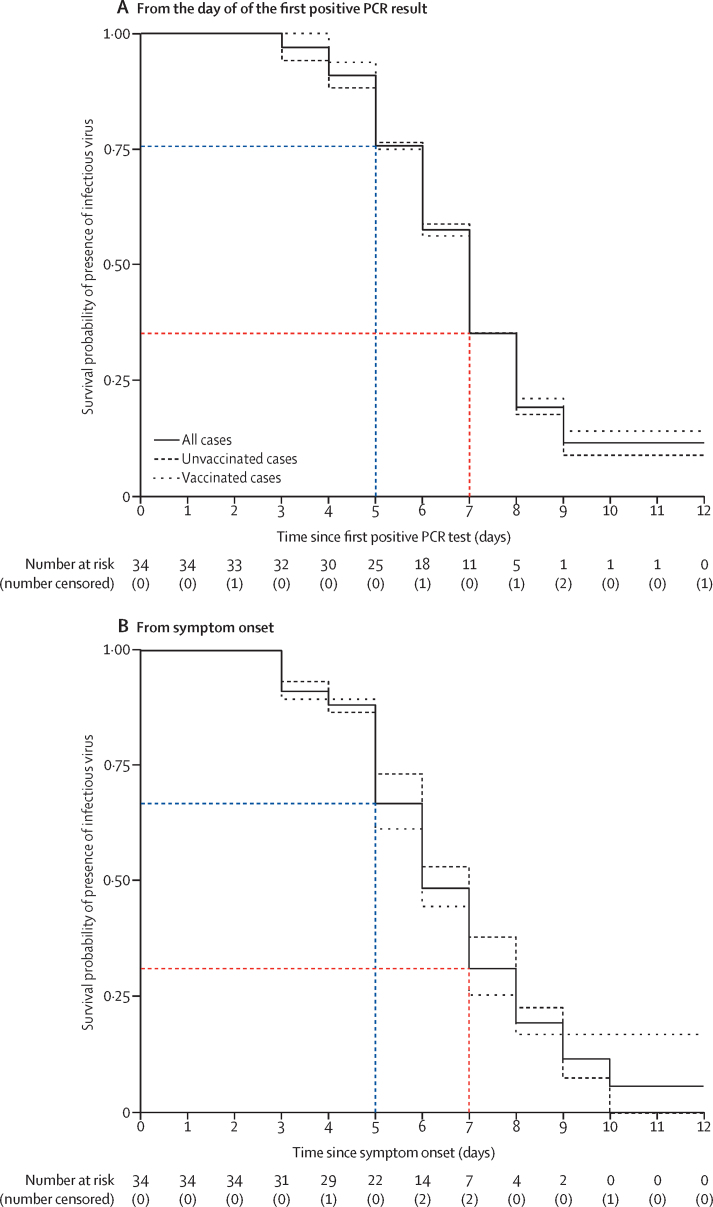
Survival probability of infectious virus presence, as determined by plaque assays Curves of the survival probability of infectious virus presence, as determined by plaque assays of cases, are plotted according to Kaplan-Meier methods. (A) Survival probability from the day of the first positive PCR result (n=34; 16 vaccinated and 18 unvaccinated). (B) Survival probability from the day of first symptom onset (n=34; 19 vaccinated and 15 unvaccinated). Curves were compared with a log-rank test. Blue lines show the cumulative count of the number of potentially infectious cases from 5 days since the first positive PCR result or first symptom onset. Red lines show the cumulative count of the number of potentially infectious cases from 7 days since first positive PCR or first symptom onset. For panel A, 23 cases were excluded entirely from the analysis for the following reasons: toxicity of the viral transport medium against Vero E6 cells (figure 3, plots 25 and 29); not shedding virus capable of forming PFUs (figure 3, plots 14, 18, 23, and 57); being early prevalent cases (figure 3, plots 12, 15, 17, 20, 21, 24, 26, 27, 33, 36, 38, 41, 45, 50, 52, and 54); and having only one PFU positive timepoint (figure 3, plot 16). For panel B, 23 cases were excluded entirely from the analysis for the following reasons: being asymptomatic (figure 3, plots 21, 23, and 52); not having a symptom onset date (figure 3, plots 1, 5, 6, 7, 8, 12, 16, 20, 22, 25, 30, 32, 45, 47, 56, and 57); toxicity of the viral transport medium against Vero E6 cells (figure 3, plot 29); not shedding virus capable of forming PFUs (figure 3, plots 14 and 18); and having inadequate PFU data (figure 3, plot 33). PFU=plaque-forming unit.

In a proportion of cases (appendix p 3), we were able to assess the probability of infectious virus presence as determined by plaque assays serially from the day of first positive PCR result (figure 4A) or from the day of first symptom onset (figure 4B). 25 (74%) of 34 cases (survival probability 76%) remained potentially infectious 5 days after their first positive PCR result, and 11 (32%) of 34 cases (survival probability 35%) after 7 days (figure 4A). Similarly, 22 (65%) of 34 cases (survival probability 67%) harboured infectious virus 5 days after symptom onset and eight (24%) of 34 cases (survival probability 35%) at 7 days. We next quantified the level of infectiousness in those who still shed infectious virus at days 5 and 7. The mean log-infectious viral load relative to the peak viral load decreased by 43% on day 5 after symptom onset, and by 83% on day 7 after symptom onset (n=29 cases with symptom onset and infectious viral load data). We found no significant difference in the proportion of cases that would be released from the day of first symptom onset between the vaccinated and unvaccinated cases (p=0·81, log-rank test; figure 4B).

LFDs were done on PCR-positive samples, as well as one day before and one day after the PCR-positive result, equating to a total of 652 LFD tests (figure 3, table 2, and table 3). 574 (88%) of 652 LFDs were carried out on PCR-positive samples. Of these PCR-positive samples, 542 (94%) were cultured for plaque assays and 257 (47%) were plaque assay positive. LFDs did not give a positive result for 305 (53%) of 574 PCR-positive samples, with a sensitivity of 47% (95% CI 43–41) and specificity of 91% (82–96). When comparing LFD performance in detecting infectious virus presence as defined by PFUs, sensitivity was higher at 79% (95% CI 74–84) and specificity was 81% (73–83), thus showing that LFDs are more sensitive for infectious virus than for viral RNA.

**Table 2:** Performance of Innova LFD tests against RNA viral load determined by RT-PCR

	**Innova LFD result**	**Number PCR negative**	**Number PCR positive**	**Sensitivity**	**Specificity**	**PPV**	**NPV**
Total	Negative Positive	71/376 (19%) 7/276 (2·5%)	305/376 (81%) 269/276 (97·5%)	47% (43–51)	91% (82–96)	97% (95–99)	19% (15–23)
Before the peak and including peak viral load	Negative Positive	40/159 (25%) 3/108 (3%)	119/159 (75%) 105/108 (97%)	47% (40–54)	93% (81–99)	97% (92–99)	25% (19–33)
Post-peak viral load	Negative Positive	31/217 (14%) 4/168 (2%)	186/217 (86%) 164/168 (98%)	47% (42–52)	89% (73–97)	98% (94–99)	14% (10–20)

Data are n/N (%) or mean (95% Cl). The viral kinetic phases were determined by serial RT-PCR. LFD=lateral flow device. PPV=positive predictive value. NPV=negative predictive value.

**Table 3:** Performance of Innova LFD tests against infectious virus determined by plaque assay

	**Innova LFD result**	**Plaque assay negative**	**Plaque assay positive**	**Sensitivity**	**Specificity**	**PPV**	**NPV**
Total	NegativePositive	243/297 (82%) 56/260 (21·5%)	54/297 (18%) 204/260 (78·5%)	79% (74–84)	81% (76–86)	78% (73–83)	82% (77–86)
Before the peak and including peak viral load	NegativePositive	76/120 (63%) 9/100 (9%)	44/120 (37%) 91/100 (91%)	67% (59–75)	89% (81–95)	91% (84–96)	63% (54–72)
Post-peak viral load	NegativePositive	167/177 (94%) 47/160 (29%)	10/177 (6%) 113/160 (71%)	92% (86–96)	78% (72–83)	71% (63–78)	94% (90–97)
Post-infectious viral load	NegativePositive	147/147 (100%) 38/38 (100%)	0/147 (0%) 0/38 (0%)	NA*	79% (73–85)	NA*	NA*

Data are n/N (%) or mean (95% Cl). The viral kinetic phases were determined by serial RT-PCR. Post-infectious viral load represents tests carried out on samples post-peak viral load, which resulted in undetectable PFUs when cultured. LFD=lateral flow device. PFU=plaque-forming unit. PPV=positive predictive value. NPV=negative predictive value.

* Calculations for the sensitivity, PPV, and NPV were not applicable (NA) in the post-infectious period as the plaque assay culture was negative.

**Figure 5:**
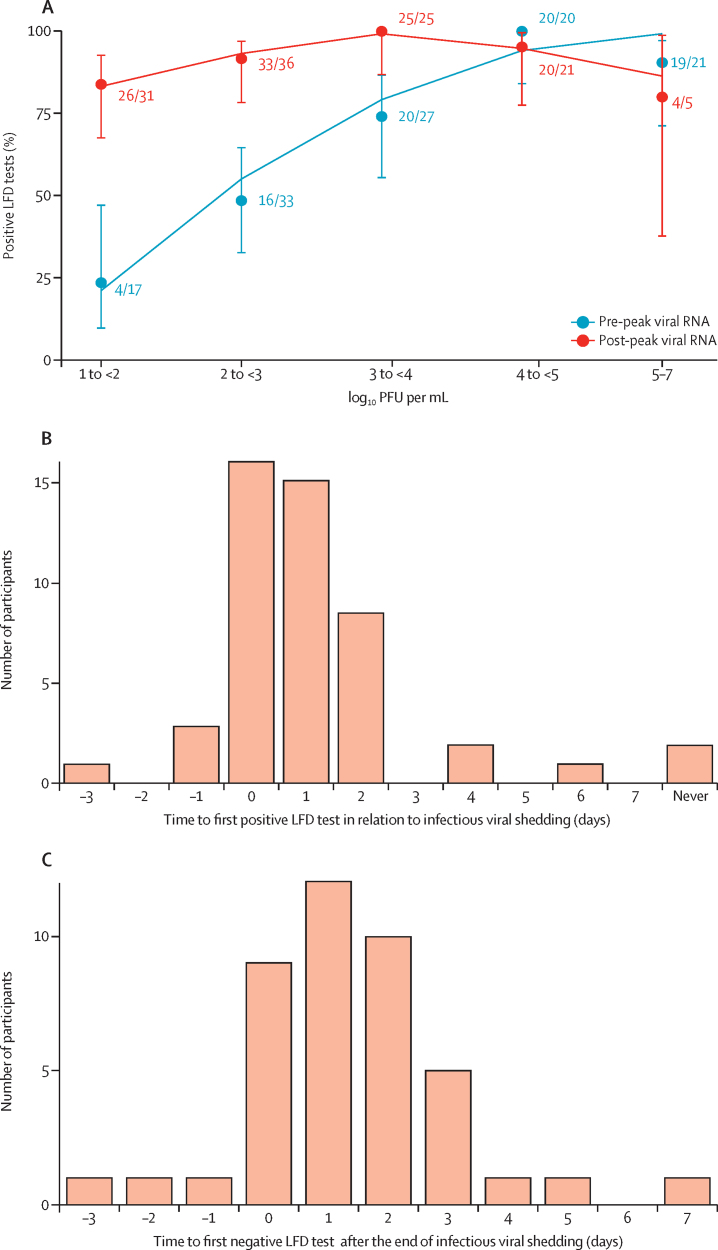
LFD reactivity in relation to the start and end of infectious viral shedding, as determined by plaque assays (A) Percentage of positive LFD tests in relation to the concentration of PFUs. Fractions indicate the sensitivity estimates for each bin. The two bins on the right were merged as only one case had a PFU concentration eligible for the last bin. Error bars represent 95% CIs. (B) Number of days taken for the LFD test to first turn positive in the presence of infectious viral shedding. (C) Number of days taken for the LFD test to first turn negative at the end of infectious viral shedding. LFD=lateral flow device. PFU=plaque-forming unit.

LFDs were negative throughout the sampling period for only two (4%) of 51 cases shedding infectious virus; both cases had sporadic, low-level infectious viral shedding (figure 3, plots 8 and 12). The sensitivity of LFDs for infectious virus changed over the course of infection (figure 2 and figure 5A), with low sensitivity during the viral growth phase and peak (67% [95% CI 59–75]). The reduced LFD sensitivity for infectious virus in the growth phase was primarily caused by false-negative results in cases with lower PFUs (figure 5A). In 17 (58%) of 29 cases in whom the LFD tested negative while the infectious virus was being shed, the LFD tested positive the following day, suggesting improved performance if used daily (figure 5B). Symptom onset preceded LFD positivity in 27 (71%) of 38 cases.

LFD sensitivity was high for infectious virus during the viral decline phase (92% [95% CI 86–96]; figure 2), with a high negative predictive value (94% [90–97]). Notably, the correlation between LFD sensitivity and PFU concentration was lost after the RNA viral load had peaked, as even cases with low PFUs after the peak were frequently detected (figure 5A). After cessation of infectious viral shedding, 38 (21%) of 185 LFDs carried out were positive (specificity 79% [73–85]). In 25 (60%) of 42 LFDs there was a lag (median 2 [IQR 1–3] days) between cessation of infectious viral shedding and conversion to LFD negativity (figure 5C).

## Discussion

In this study, we have characterised the window of infectiousness of mild COVID-19 in a real-world community setting with longitudinal empirical data. Crucially, our study allowed us to capture the onset and end of infectious viral shedding, providing a definitive estimate of the bounds of the infectious window. The 5-day median window of infectious viral shedding we delineated explains key epidemiological observations, including the marked decline in transmission within a week of symptom onset.^12^ Moreover, the heterogeneity we observed in the infectious viral load trajectories probably explains some of the variability in secondary transmission between individuals, although host, behavioural, and environmental factors also strongly influence transmission, including, in particular, the propensity for viral aerosolisation.^4^

We detected viral RNA shedding in over half of the cases before the onset of symptoms and it has hitherto been assumed that the presence of presymptomatic viral RNA shedding implied the presence of infectious virus.^13^, ^14^_._ However, we found that infectious viral shedding commenced before the onset of symptoms in only 25% or less of cases, contradicting modelling studies.^15^ Although our sample size was small, our findings were based on daily quantitative viral culture and daily symptom records in real-world community contacts, making it likely that the temporal relationship we observed between onset of symptoms and onset of infectious viral shedding is generalisable.

Given the societal, psychological, and economic costs of self-isolating for longer than is necessary, our empirical data from community contacts could inform new guidance to minimise self-isolation periods to match the duration of infectiousness. Many national public health agencies have recently changed guidance to shorten self-isolation periods based largely on modelling^16^ or qualitative cross-sectional viral culture data.^17^ However, our empirical data suggest that a crude 5-day self-isolation period releases two-thirds of still-infectious cases into the community, albeit with a 43% reduction in mean log-infectious viral load relative to peak viral load, whereas by 7 days post-symptom onset, one-third are still infectious with an 83% reduction in infectious viral load. Such evidence could enable policy makers, and the public, to calibrate self-isolation guidance.

We observed that the growth rate of infectious viral load and RNA viral load correlated positively with their respective peaks and negatively with their respective decline rates. Individual infections with the fastest growth rates thus have the slowest viral clearance, suggesting that the potency of the early mucosal innate immune response shapes the subsequent course of infection. The progressive 100-times decline in SARS-CoV-2 infectious virions produced per viral RNA copy over the course of infection also implicates adaptive host responses that neutralise the infectivity of the virus. Cross-sectional studies often assume a constant ratio between RNA viral load and infectiousness, but our data indicate that future outbreak investigations and epidemiological studies should consider the timing of sample collection relative to the course of infection when estimating risk of transmission from RNA viral load.^18^, ^19^

There was notable inter-individual consistency in the upper limit of RNA viral load and infectious viral load attained, which was similar to the corresponding peak RNA viral load and peak infectious viral load observed in the recent controlled human infection challenge model (CHIM).^5^ However, the overall distribution of the peak values in our cohort was somewhat wider than that of CHIM, probably reflecting our more demographically heterogeneous community cases as well as variability in the infecting dose, and route, for transmission events in the community compared with the standardised dose of wild-type virus (SARS-CoV-2/human/GBR/484861/2020) inoculated directly into the nares of pre-selected SARS-CoV-2-naive healthy volunteers in CHIM.

Our study provided the opportunity to rigorously test whether LFDs align more with infectious viral shedding than PCR positivity. LFD sensitivity was poor during the viral growth phase, especially at lower PFU concentrations, which is crucial for early diagnosis. During the decline phase, LFD sensitivity against viral culture was much higher than during the growth phase regardless of PFU concentration, with a median 2-day lag in LFD positivity after cessation of infectious viral shedding. Prolonged LFD positivity in the absence of evidence for active viral replication probably reflects persisting viral antigens from remnants of infected cells as seen with RT-PCR with lingering viral RNA fragments.^20^ The increase in LFD sensitivity with PFU concentration over time during the growth phase could reflect the time taken for viral antigens to be produced in sufficient quantity to be detectable by LFDs.

Our findings provide a rationale for using LFDs to safely accelerate deisolation, as embodied in certain policies^21^ and supported by previous modelling, but caution against the use of LFDs for initial SARS-CoV-2 diagnosis unless used daily. Moreover, symptom onset occurring soon after known exposure should not be ignored even if accompanied by negative LFD results. The few previous studies that attempted to link individual-level LFD, PCR, and viral culture data reported LFD sensitivity against culturable virus to be 93–98%,^4^, ^22^ and did not account for when in the course of infection samples were taken. Diagnostic performance during the early phase of infection, when most transmission occurs, was therefore not specifically assessed, resulting in controversial overestimates of LFD sensitivity.^3^

Our small cohort size rendered statistical comparison between subgroups underpowered and this was not the primary objective of our study. Notwithstanding, since approximately half of our cases were vaccinated before exposure, we took the opportunity to compare them with unvaccinated cases in an exploratory analysis. The unvaccinated cases were infected with pre-alpha, alpha, and delta variants, whereas all vaccinated cases were delta variant-infected, which could confound comparison if viral load kinetics differ between different strains.^9^, ^23^, ^24^ Overall, we found that the decline rate of infectious viral shedding was somewhat faster in vaccinated cases, consistent with the observation that vaccination is associated with faster clearance of viral RNA in larger studies.^9^ Given our novel fundamental observation that infectious viral load decline rate is strongly inversely correlated with both infectious viral load growth rate and peak infectious viral load, the faster decline in vaccinated cases suggests that vaccination is associated with slower infectious viral load growth rate and lower peak infectious viral load. This prediction was recently corroborated by the empirical observation that vaccinated breakthrough cases of delta variant infection have significantly lower infectious viral load than do unvaccinated delta-infected cases.^24^

Our study has several limitations. Very young and older age groups, with their attendant risk factors for severe illness and hospitalisation, were under-represented in our cohort. However, our cohort does reflect the population and setting responsible for most SARS-CoV-2 transmission globally. URT samples were self-performed by study participants, which can result in variable sensitivity and specificity.^25^ LFDs were carried out by trained laboratory staff, but their performance depends on the test operator and individuals in a community setting have been found to perform worse than laboratory personnel.^26^ Different commercial products vary in their diagnostic accuracy and in their performance for detecting different variants and we only evaluated the Innova LFD.

The timeframe of our study precluded analysis of the omicron variant, which became prevalent at the end of 2021. Although some recent studies suggest that omicron infections have lower RNA viral load compared with delta infections,^27^, ^28^ others suggest that the RNA viral load is similar.^29^, ^30^ Notably, the single study that compared infectious viral loads found that it was lower for omicron than delta infections, but did not define the infectious window.^24^ Another study found that the duration of viral RNA shedding was 10% shorter for omicron than delta variants,^27^ suggesting that the omicron infectious window might likewise be slightly shorter, although it has not yet been determined. Strategies based on our infectious window would therefore be, if anything, slightly cautious when applied to omicron, consistent with the principle of public health infection control that favours caution over risk.

Given that no reference standard for infectiousness exists, our data should be interpreted by policy makers as defining the window of potential infectiousness, because the presence of infectious virus in the URT does not inevitably lead to secondary transmission, which depends on several other host, behavioural, and environmental factors, including the propensity for viral aerosolisation.^31^ Conversely, a lack of cultivability might not always mean a lack of infectiousness because mammalian cell lines in vitro (as used in the plaque assay) and human airway epithelial cells in vivo might differ in their permissiveness for viral infection and growth. Notwithstanding, PFU concentration from URT swabs, as measured here, indicates potential infectiousness at a given point in time, as supported by the dose–response relationship between infectious virus dose quantified by in vitro cell culture and the likelihood of clinical infection in SARS-CoV-2 animal challenge models^32^ and the human influenza challenge model.^33^

In conclusion, we defined the infectious window and dynamics of SARS-CoV-2 infectiousness and its inter-individual variability. Preliminary evidence from our study has already informed policy^21^ and the real-world evidence presented here could be used to improve infection control policies and optimise guidance on self-isolation to minimise secondary transmission.

**This online publication has been corrected. The corrected version first appeared at thelancet.com/respiratory on September 5, 2022**

## Data sharing

An anonymised, de-identified version of the dataset can be made available upon request to the corresponding author to allow all results to be reproduced. Modelling code will also be made publicly available on the GitHub repository.

## Declaration of interests

NF reports grants from the UK Medical Research Council, UK National Institute of Health and Care Research (NIHR), UK Research and Innovation, Community Jameel, Janssen Pharmaceuticals, Bill & Melinda Gates Foundation, and Gavi, the Vaccine Alliance; consulting fees from the World Bank; payment or honoraria from the Wellcome Trust; travel expenses from WHO; advisory board participation for Takeda; and is a senior editor of the *eLife* journal. All other authors declare no competing interests.
