# Supplementary material for: Onset and window of SARS-CoV-2 infectiousness and temporal correlation with symptom onset: a prospective, longitudinal, community cohort study
Source: Lancet Respir Med. 2022 Nov;10(11):1061–73. doi: 10.1016/S2213-2600(22)00226-0 (PMC9388060; doi:10.1016/S2213-2600(22)00226-0)
Supplement: Supplementary appendix [file mmc1.pdf]

THE LANCET
Respiratory Medicine

Supplementary appendix

This appendix formed part of the original submission and has been peer reviewed. We post it as supplied by the authors.

Supplement to: Hakki S, Zhou J, Jonnerby J, et al. Onset and window of SARS-CoV-2 infectiousness and temporal correlation with symptom onset: a prospective, longitudinal, community cohort study. *Lancet Respir Med* 2022; published online Aug 18. https://doi.org/10.1016/S2213-2600(22)00226-0.

Supplementary Information

Supplementary Tables

Table S1: Table depicting the demographic characteristics of unvaccinated cases infected with delta, alpha and pre-alpha strains and the vaccinated PCR-positive cases infected with the delta variant. **Page 2**

Table S2: Table summarising the number of cases within each analysis and the reasons for case exclusion. **Page 3**

Table S3: Posterior probability distributions of the group-level parameters fitted to the RNA viral load trajectories. **Page 4**

Table S4: Posterior probability distributions of the group-level parameters fitted to infectious virus shedding (plaque forming units) trajectories. **Page 5**

Table S5: Table depicting the p-values of the t-test used to evaluate correlations between participants age and the median values of their kinetic parameters for both the RNA viral (copy numbers) and infectious virus (PFU) trajectories. **Page 5**

Table S6: Summary statistics for Bayesian hierarchical modelled viral kinetics stratified by vaccination status, derived from RNA viral load data and plaque assays. **Page 6**

Extended Data Figures:

Fig. S1: Innova lateral flow devices showing the different grades for SARS-CoV-2 detection. **Page 7**

Fig.S2: Box and whisker plots showing the peak viral RNA copies and plaque forming units. **Page 8**

Fig.S3: Bayesian hierarchical model fits of SARS-CoV-2 viral dynamics captured through daily sampling for unvaccinated (green) and vaccinated cases (purple). **Page 9**

Fig.S4: The relationship between RNA viral load and infectious viral load (PFUs) over time since the first study PCR-positive result. **Page 10**

Supplementary Methods

Study recruitment. **Page 11**

Plaque assays. **Page 11**

Lateral flow devices. **Page 12**

Viral whole genome sequencing for lineage assignments. **Page 12**

Modelling viral kinetics. **Page 12-14**

Supplementary methods references. **Page 14-15**

Supplementary Tables

Table S1: Table depicting the demographic characteristics of unvaccinated cases infected with delta, alpha and pre-alpha strains and the vaccinated PCR-positive cases infected with the delta variant. Fisher's Exact tests were performed to determine differences for each characteristic between unvaccinated and fully vaccinated cases. *The delta unvaccinated cohort was significantly younger than the alpha and pre-alpha unvaccinated cases (p=0.002). †Body mass index (BMI) is missing for children. ‡Health conditions included asthma (n=5), chronic obstructive pulmonary disease (n=1), hepatitis B (n=1), ischemic heart disease (n=2), type 2 diabetes (n=2), moderate liver disease (n=1), second trimester of pregnancy (n=1). §Index symptom onset was used as a proxy for exposure.

**Characteristics**		**All cases**	**Pre-alpha**	**Alpha**	**Unvaccinated delta**	**All unvaccinated cases***	**Delta vaccinated cases**	**P-value**
		**(n = 57)**	**(n = 13)**	**(n = 12)**	**(n = 7)**	**(n = 32)**	**(n = 25)**	
**Sex**	Female (%)	34 (60)	7 (54)	9 (75)	2 (29)	18 (56)	16 (64)	
	Male (%)	23 (40)	6 (46)	3 (25)	5 (71)	14 (44)	9 (36)	0.60
	Unknown (%)	0 (0)	0 (0)	0 (0)	0 (0)	0 (0)	0 (0)	..
**Age in years**	Median (IQR)	41 (29 - 49)	32 (27 - 46)	47 (31 - 50)	15 (13 - 24)	33 (25 - 47)	44 (41 - 49)	
	<18 (%)	7 (12)	0 (0)	1 (8)	5 (71)	6 (19)	1 (4)	
	18-49 (%)	40 (70)	11 (85)	8 (67)	2 (29)	21 (66)	19 (76)	
	50-64 (%)	10 (18)	2 (15)	3 (25)	0 (0)	5 (16)	5 (20)	
	≥ 65 (%)	0 (0)	0 (0)	0 (0)	0 (0)	0 (0)	0 (0)	0.32
**BMI†**	Median (IQR)	25.2 (21.2 - 28.8)	27.9 (24 - 31.8)	27.4 (24.9 - 30.4)	19.7 (16.8 - 20.6)	26 (20.6 - 30.2)	24.3 (21.9 - 28.2)	..
	Underweight (%)	4 (7)	0 (0)	1 (8)	2 (29)	3 (9)	1 (4)	
	Normal (%)	23 (40)	4 (31)	2 (17)	5 (71)	11 (34)	12 (48)	
	Overweight (%)	17 (30)	3 (23)	5 (42)	0 (0)	8 (25)	9 (36)	
	Obese (%)	5 (9)	2 (15)	2 (17)	0 (0)	4 (13)	1 (4)	
	Morbidly obese (%)	6 (11)	3 (23)	2 (17)	0 (0)	5 (16)	1 (4)	0.37
	Unknown (%)	2 (4)	1 (8)	0 (0)	0 (0)	1 (3)	1 (4)	..
**Ethnicity**	White (%)	51 (89)	12 (92)	11 (92)	6 (86)	29 (91)	22 (88)	
	Non-white (%)	6 (11)	1 (8)	1 (8)	1 (14)	3 (9)	3 (12)	1.0
	Unknown (%)	0 (0)	0 (0)	0 (0)	0 (0)	0 (0)	0 (0)	..
**Comorbidities or pregnancy‡**	Yes (%)	13 (23)	3 (23)	4 (33)	1 (14)	8 (25)	5 (20)	
	No (%)	44 (77)	10 (77)	8 (67)	6 (86)	24 (75)	20 (80)	0.78
**Smoking status**	Current (%)	6 (11)	1 (8)	2 (17)	0 (0)	3 (9)	3 (12)	
	Former (%)	6 (11)	3 (23)	0 (0)	0 (0)	3 (9)	3 (12)	
	Never (%)	44 (77)	9 (69)	10 (83)	6 (86)	25 (78)	19 (76)	1.0
	Unknown (%)	1 (2)	0 (0)	0 (0)	1 (14)	1 (3)	0 (0)	..
**Type of exposure**	Household (%)	54 (95)	10 (77)	12 (100)	7 (100)	29 (91)	25 (100)	
	Non-household (%)	3 (5)	3 (23)	0 (0)	0 (0)	3 (9)	0 (0)	0.25
**Days between second vaccination and Exposure§**		..	..	..	..	..	96 (79-116)	..

Table S2: Table summarising the numbers of cases within each analysis and the reasons for case exclusion.

Analysis	Exclusion
Longitudinal quantification of infectious SARS-CoV-2 through the course of infection and its relationship to viral RNA load	
**Characterising the window of infectiousness**<br>(n=42; 15 cases excluded)	2 cases excluded due to the toxicity of the viral transport media used for these contacts against Vero E6 cells (see Methods)<br>• Fig.2, plots 25 & 29<br>4 cases excluded due to not shedding virus capable of forming PFUs<br>• Fig.2, plots 14, 18, 23 & 57<br>2 cases excluded due to shedding infectious virus from the study start<br>• Fig.2, plots 12 & 45<br>7 cases excluded as they continued to shed infectious virus at the end of the study<br>• Fig.2, plots 4, 9, 31, 36, 46, 48 & 50
Temporal relationship of infectious virus shedding with symptom onset	
**Symptom onset in relation to the onset of viral RNA shedding and peak viral RNA shedding**<br>(n=38; 19 cases excluded)	3 cases excluded as they were asymptomatic<br>• Fig.2, plots 21, 23 & 52<br>16 cases excluded as they did not have a symptom onset date<br>• Fig.2, plots 1, 5, 6, 7, 8, 12, 16, 20, 22, 25, 30, 32, 45, 47, 56, 57
**Symptom onset in relation to the onset of infectious viral shedding and peak infectious viral shedding**<br>(n=35; 22 cases excluded)	As above and:<br>2 of the remaining cases excluded due to not shedding virus capable of forming PFUs<br>• Fig.2, plots 14 & 18<br>1 of the remaining cases excluded due to the toxicity of the viral transport media used for these contacts against Vero E6 cells (see Methods)<br>• Fig.2, plot 29
Growth rate of infectious viral shedding predicts decline rate	
**Bayesian hierarchical model fits of infectious viral shedding**<br>(n=47; 10 cases excluded from the cohort of 57)	2 cases excluded due to the toxicity of the viral transport media used for these contacts against Vero E6 cells (see Methods)<br>• Fig.2, plots 25 & 29<br>4 cases excluded due to not shedding virus capable of forming PFUs<br>• Fig.2, plots 14, 18, 23 & 57<br>4 cases excluded due to not having enough PFU data during the decline phase for modelling<br>• Fig.2, plots 36, 46, 48 & 50
**The ratio of RNA copies/ml to PFU/ml during the course of infection**<br>(n=49, 8 cases excluded)	2 cases excluded due to the toxicity of the viral transport media used for these contacts against Vero E6 cells (see Methods)<br>• Fig.2, plots 25 & 29<br>4 cases excluded due to not shedding virus capable of forming PFUs<br>• Fig.2, plots 14, 18, 23 & 57<br>2 cases excluded due to prolonged infectious virus shedding likely due to host factors<br>• Fig.2, plots 46 & 48
Effect of shortening isolation periods on the proportion of cases released whilst still infectious	
**The probability of infectious virus presence from the day of first PCR-positive result**<br>(n=34; 23 cases excluded from the cohort of 57)	2 cases excluded due to the toxicity of the viral transport media used for these contacts against Vero E6 cells (see Methods)<br>• Fig.2, plots 25 & 29<br>4 cases excluded due to not shedding virus capable of forming PFUs<br>• Fig.2, plots 14, 18, 23 & 57<br>16 of the remaining early prevalent cases excluded<br>• Fig.2, plots 12, 15, 17, 20, 21, 24, 26, 27, 33, 36, 38, 41, 45, 50, 52 & 54<br>1 of the remaining cases excluded for having only 1 PFU positive timepoint<br>• Fig.2, plot 16
**The probability of infectious virus presence from the day of first symptom onset**<br>(n=34; 23 cases excluded from the cohort of 57)	3 cases excluded as they were asymptomatic<br>• Fig.2, plots 21, 23 & 52<br>16 cases excluded as they did not have a symptom onset date<br>• Fig.2, plots 1, 5, 6, 7, 8, 12, 16, 20, 22, 25, 30, 32, 45, 47, 56, 57<br>1 of the remaining cases excluded due to the toxicity of the viral transport media used for these contacts against Vero E6 cells (see Methods)<br>• Fig.2, plot 29<br>2 of the remaining cases excluded for not shedding virus capable of forming PFUs<br>• Fig.2, plots 14 & 18<br>1 of the remaining cases excluded for having an inadequate amount of PFU data<br>• Fig.2, plot 33

Table S3: Posterior probability distributions of the group-level parameters fitted to the RNA viral load trajectories. The results for the sensitivity analysis, where vaccinated and unvaccinated individuals were combined in a single group and only including participants with at least 5 positive PFU samples (see Extended Data Fig.3), confirmed the findings from the full cohort while showing better model convergence overall. See the Methods section for parameter definitions. Abbreviations: Crl, credible intervals.

	Full cohort (n=57)			Sensitivity analysis (n=29)		
Parameter	Median (95 % Crl)	$\hat{R}$	n_{eff}	Median (95 % Crl)	$\hat{R}$	n_{eff}
μ_{11}	6.54 (6.21, 6.86)	1.0	7258	6.54 (6.21, 6.86)	1.00	4798
μ_{12}	7.01 (6.66, 7.37)	1.01	6232	..	..	..
μ_{21}	1.64 (1.31, 1.98)	1.0	5631	1.64 (1.31, 1.98)	1.01	1692
μ_{22}	1.68 (1.32, 2.05)	1.01	1179	..	..	..
μ_{31}	0.32 (0.11, 0.52)	1.01	4762	0.32 (0.11, 0.52)	1.00	2157
μ_{32}	0.66 (0.43, 0.88)	1	5967	..	..	..
δ_1	1.86 (1.46, 2.41)	1.01	925	1.86 (1.46, 2.41)	1.00	5101
δ_2	0.89 (0.71, 1.14)	1.01	3087	0.89 (0.71, 1.14)	1.01	3444
δ_3	0.54 (0.43, 0.68)	1.01	2831	0.54 (0.43, 0.68)	1.00	4461
c_{12}	0.15 (-0.16, 0.43)	1.01	1581	0.15 (-0.16, 0.43)	1.01	5478
c_{13}	-0.12 (-0.41, 0.20)	1.01	870	-0.12 (-0.41, 0.20)	1.00	6273
c_{23}	-0.34 (-0.57, -0.06)	1.0	14726	-0.34 (-0.57, -0.06)	1.00	6545
σ_v	1.62 (1.52, 1.74)	1.03	85	1.62 (1.52, 1.74)	1.04	102
p	0.05 (0.04, 0.07)	1.06	40	0.05 (0.04, 0.07)	1.00	6158
x_0	0.19 (-3.09, 2.84)	1.03	107	0.19 (-3.09, 2.84)	1.00	6356
σ_0	7.96 (6.09, 10.42)	1.07	35	7.96 (6.09, 10.42)	1.01	903

Table S4: Posterior probability distributions of the group-level parameters fitted to the infectious virus shedding (plaque forming units) trajectories. The results for the sensitivity analysis, where vaccinated and unvaccinated individuals were included in a single group and only including participants with at least 5 positive PFU samples (see Extended Data Fig.3), confirmed the findings from the full cohort while showing better model convergence overall. See the Methods section for parameter definitions. Abbreviations: Crl, credible intervals.

	Full cohort (n=47)			Sensitivity analysis (n=29)		
Parameter	Median (95 % Crl)	$\hat{R}$	n_{eff}	Median (95 % CrI)	$\hat{R}$	n_{eff}
μ_{11}	4.43 (3.86 4.96)	1.01	5281	3.79 (3.42, 4.15)	1.0	2558
μ_{12}	5.40 (4.74 6.03)	1.01	4514	..	..	..
μ_{21}	1.63 (1.20, 2.06)	1	5755	1.52 (1.23, 1.84)	1.0	2338
μ_{22}	1.67 (1.26, 2.14)	1.03	147	..	..	..
μ_{31}	0.74 (0.47, 1.01)	1	1569	0.96 (0.67, 1.27)	1.0	1990
μ_{32}	1.50 (1.13, 1.81)	1	76	..	..	..
δ_1	3.12 (2.47, 4.01)	1.05	138	2.08 (1.57, 2.87)	1.0	3730
δ_2	0.87 (0.65, 1.20)	1.0	5483	0.75 (0.54, 1.05)	1.0	3623
δ_3	0.57 (0.44, 0.76)	1.01	6125	0.75 (0.56, 1.03)	1.0	3357
c_{12}	0.36 (-0.06, 0.66)	1.01	5524	0.44 (0.06, 0.72)	1.0	5347
c_{13}	0.26 (-0.17, 0.62)	1.06	49	0.32 (-0.07, 0.63)	1.0	5682
c_{23}	-0.46 (-0.71, -0.15)	1.01	4532	-0.68 (-0.85, -0.43)	1.0	4584
σ_v	1.07 (1.00, 1.21)	1.32	10	1.19 (1.05, 1.36)	1.0	2122
p	0.04 (0.03, 0.06)	1.07	37	0.05 (0.03, 0.08)	1.0	4422
x_0	-0.19 (-1.73, 1.37)	1.07	39	0.18 (-1.02, 1.25)	1.0	5870
σ_0	2.54 (1.25, 5.55)	1.56	7	2.21 (1.08, 3.80)	1.0	2051

Table S5: Table depicting the p-values of the t-test used to evaluate correlations between participants' age and the median values of their kinetic parameters for both the RNA viral (copy numbers) and infectious virus (PFU) trajectories. Abbreviations: PFU, plaque forming unit. R-squared values were calculated for correlations where p<0.05.

RNA trajectory						
	p-values			R-squared		
	Growth rate	Decline rate	Peak	Growth rate	Decline rate	Peak
Age	0.23	0.34	0.81	..	..	..
Sex	0.04	0.68	0.76	0.06	..	..
BMI	0.27	0.30	0.21	..	..	..
PFU trajectory						
	p-values			R-squared		
	Growth rate	Decline rate	Peak	Growth rate	Decline rate	Peak
Age	0.21	0.29	0.41	..	..	..
Sex	0.74	0.83	0.44	..	..	..
BMI	0.96	0.005	0.07	..	0.15	..

Table S6: Summary statistics for Bayesian hierarchical modelled viral kinetics stratified by vaccination status, derived from RNA viral load data and plaque assays. Within-sample posterior mean estimates given, with 95% credible intervals in parentheses. Viral RNA shedding (copies/ml) n=57; 32 unvaccinated, 25 vaccinated. Infectious virus shedding (PFU) n=47; 27 unvaccinated, 20 vaccinated. *All vaccinated cases were delta-infected. Unvaccinated cases include 13 cases infected with pre-alpha, 12 alpha and 7 delta variants of SARS-CoV-2. 10 cases were excluded entirely from the infectious virus shedding (PFU) Bayesian hierarchical modelling due to (i) the toxicity of the viral transport media used for these contacts against Vero E6 cells (see Methods); Fig.2, plots 25 & 29, (ii) not shedding virus capable of forming PFUs; Fig.2, plots 14, 18, 23 & 57 and (iii) to not having enough PFU data during the decline phase for modelling; Fig.2, plots 36, 46, 48 & 50. †Bayes factors were estimated by BF = pp/(1-pp), where pp is the posterior probability that the within-sample mean of the parameter is greater for vaccinated than unvaccinated cases. Abbreviations: PFU, plaque forming unit; Crl, credible intervals.

		**Viral RNA shedding (copies/ml) (n=57; 32 unvaccinated, 25 vaccinated)**		**Infectious virus shedding (PFU) (n=47; 27 unvaccinated, 20 vaccinated)**	
		**Median (Crl 95%)**	***Bayes factor†***	**Median (Crl 95%)**	***Bayes factor†***
**Peak VL/PFU:** Log10 RNA/copies per ml or PFU/copies per ml	Unvaccinated	8.3 (6.4,9.6)	1.8	4.6 (1.4, 6.6)	2
	Vaccinated*	8.5 (7.2 9.7)		4 (3.3, 7.3)	
**Total amount of virus shed:** Area under the curve of trajectory	Unvaccinated	55 (20, 119)	1.2	9.2 (0.0, 21.0)	0.8
	Vaccinated	48 (10, 74)		31 (14, 66)	
**Exponential growth rate:** E-foldings per day	Unvaccinated	4.9 (1.3, 36.0)	1.1	5.2 (1.1, 25.9)	0.9
	Vaccinated	4.8 (1.5, 35.4)		4.4 (1.7, 34.5)	
**Exponential decline rate:** E-foldings per day	Unvaccinated	1.6 (0.5, 5.3)	1.9	2.1 (0.6, 5.0)	4.1
	Vaccinated	1.8 (0.9, 5.8)		4.7 (1.3, 12.9)	
**Growth phase duration:** Days	Unvaccinated	3.5 (0.5, 14.8)	1.0	1.4 (0.2, 5.3)	1.4
	Vaccinated	3.6 (0.5, 9.8)		2.0 (0.4, 4.6)	
**Decline phase duration:** Days	Unvaccinated	10.6 (2.7, 29.3)	0.5	3.2 (0.3, 7.1)	0.5
	Vaccinated	9.3 (2.5, 17.4)		1.9 (0.8, 7.4)	

Supplementary Figures

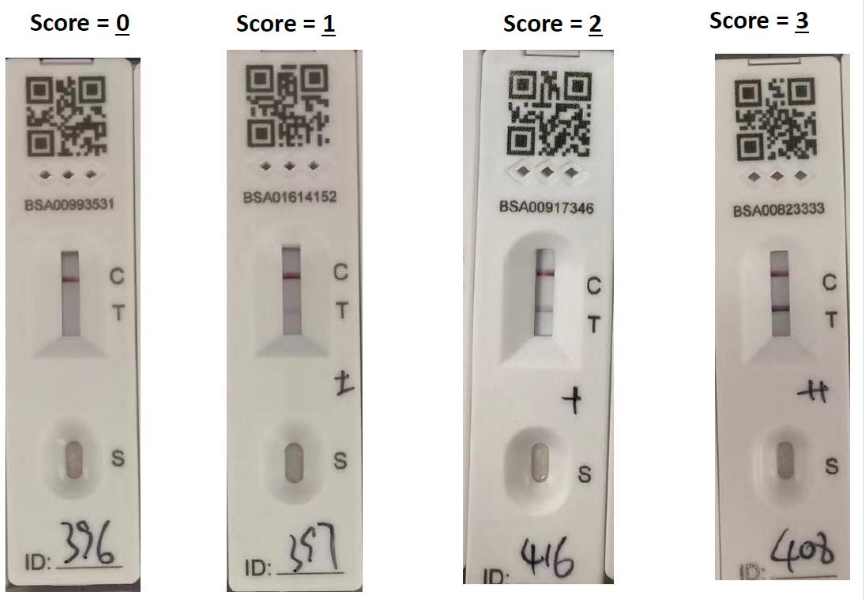

Fig.S1: Innova lateral flow devices showing the different grades for SARS-CoV-2 detection. Grade 0 (negative) indicated with no band shown for the letter T (test). Grade 1 (weak positive) indicated a with a subtle band shown for the T. Grade 2 (moderate positive) indicated with a band shown for T, but not as dense as the line for the letter C (control). Grade 3 (strong positive) indicated with the band for T appearing similar to the band for C. No bands or only one band next to T would indicate that the test is void.

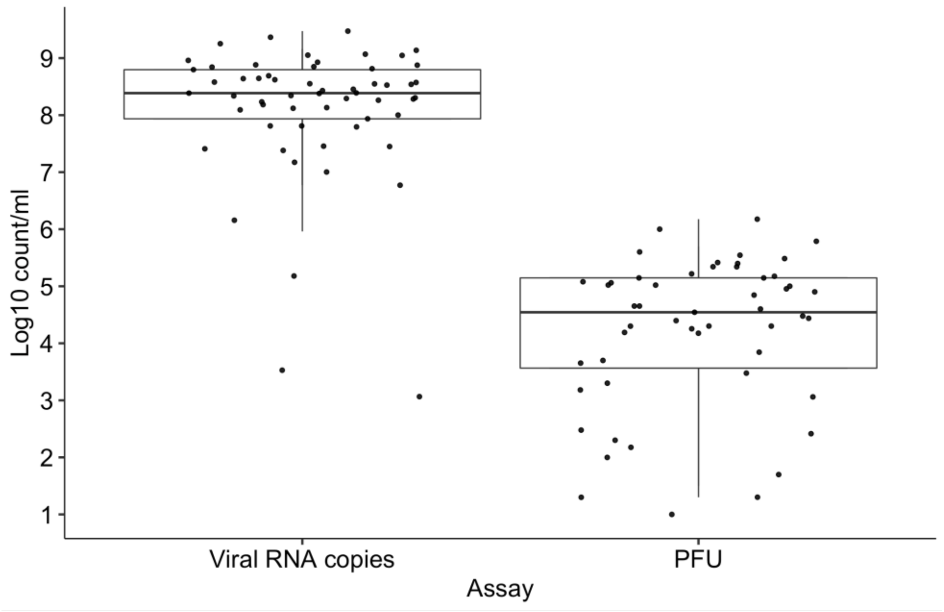

Fig.S2: Box and whisker plots showing the peak viral RNA copies and plaque forming units. The line within the box shows the median, the edges of the box show the interquartile range, and the whiskers show the 95% confidence interval. n=57 cases for peak viral RNA copies, n=51 cases for peak PFU. Abbreviations: PFU, plaque forming units.

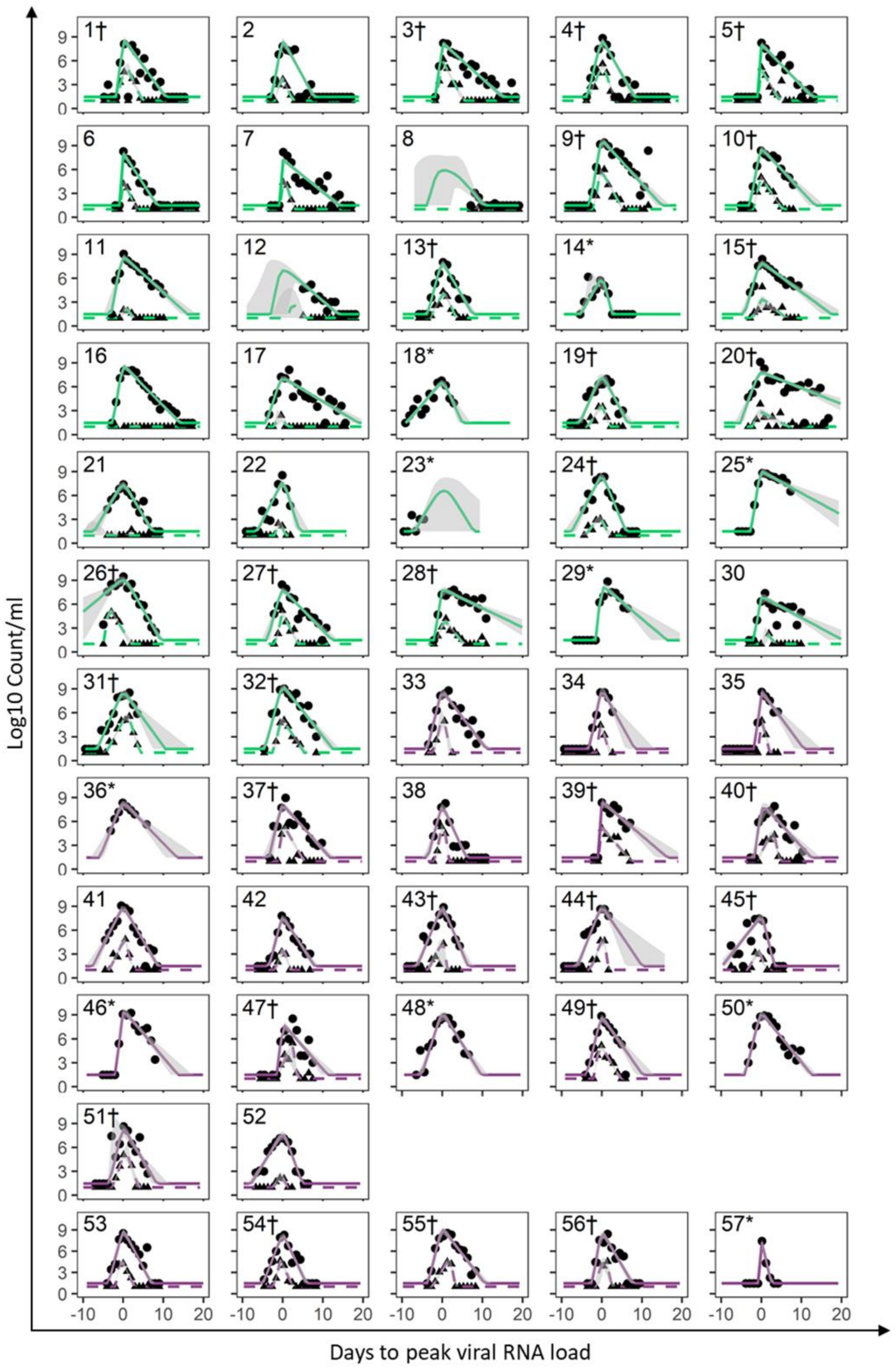

Fig.S3: Bayesian hierarchical model fits of SARS-CoV-2 infectious viral load and RNA viral dynamics captured through daily sampling for unvaccinated (green) and vaccinated cases (purple). Dots show viral load in units of log10 copies per ml and the solid lines represent the modelled trajectory. Triangles show plaque forming units in log10 per ml and the dashed lines represent median modelled trajectory. Grey ribbons represent 95% credible intervals. *10/57 of the cases were excluded from Bayesian hierarchical model fits on their PFU trajectories due to (i) the toxicity of the viral transport media used for these contacts against Vero E6 cells (see Methods); plots 25 and 29, (ii) not shedding virus capable of forming PFUs; plots 14, 18, 23 and 57 and (iii) not having enough PFU data during the decline phase for modelling; plots 36, 46, 48 and 50 plots. Plots marked with daggers (†) indicate trajectories included in the sensitivity analysis (with at least five positive PFU samples through the course of infection).

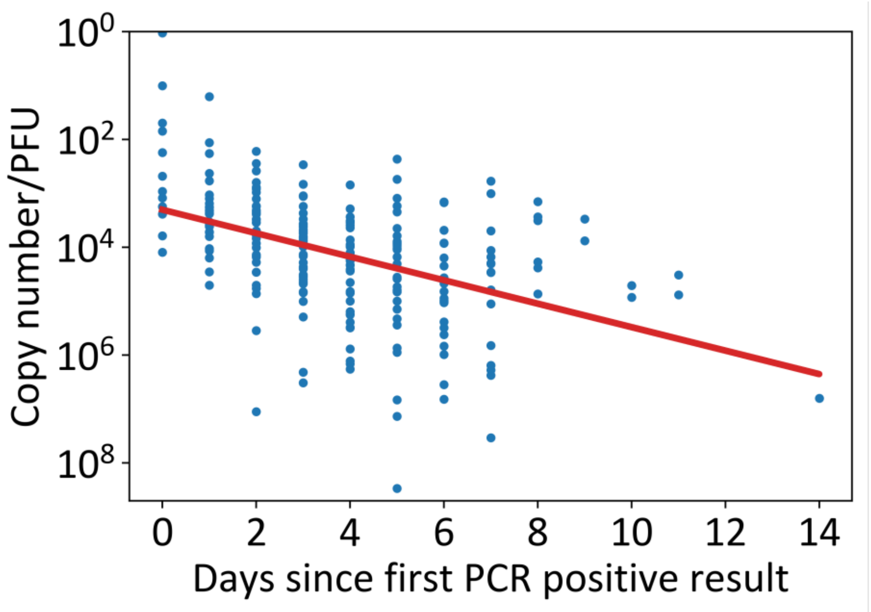

Fig.S4: The relationship between RNA viral load and infectious viral load (PFUs) over time since the first study PCR-positive result. The line of best fit is indicated with a red line. The Y axis scale is inverted to show the viability of virions attenuating over the course of infection. n=22 vaccinated cases, 101 datapoints, n=27 unvaccinated cases, 137 datapoints. 8 cases were excluded entirely from the analysis due to (i) the toxicity of the viral transport media used for these contacts against Vero E6 cells (see Methods); Fig.2, plots 25 & 29, (ii) not shedding virus capable of forming PFUs; Fig.2, plots 14, 18, 23 & 57 and (iii) prolonged infectious virus shedding likely due to host factors; Fig.2, plots 46 & 48.

Supplementary Methods

Study Recruitment

Close contacts of newly PCR-positive index cases were notified to the UK contract tracing system (National Health Service Test and Trace) and invited to participate if they were (i) 5 years and older, (ii) within 5 days of the index case's symptom onset and (iii) could provide informed consent. Recruited contacts self-performed combined nose (anterior nares) and throat swabs (hereafter denoted upper respiratory tract [URT] swabs) at home after detailed instruction by study nurses, for up to 20 consecutive days.

ATACCC enrolment spanned two separate time periods: ATACCC1 enrolled 393 contacts from 327 households from 13th September 2020 to 31st March 2021 during the SARS-CoV-2 pre-alpha and alpha variant waves while ATACCC2 enrolled 345 contacts from 215 households from 24th May 2021 to October 28th 2021 during the delta variant wave.

During enrolment, demographic information was collected using questionnaires. Vaccination records were obtained from GP records with consent. Unvaccinated cases were defined as those who had not received any COVID-19 vaccination prior to index symptom onset, our proxy for exposure. Fully vaccinated cases were defined as those who had received their second COVID-19 vaccination ≥14 days prior to index symptom onset. Cases who received only one dose of vaccine prior to index symptom onset were excluded. Due to the timing of our study recruitment respective to the UK vaccine rollout, none of our vaccinated contacts had more than two vaccinations.

Plaque assays

3ml of viral transport media (VTM) (2 brands: Copan Universal Transport Medium System, Copan Diagnostics; VTM2, MANTACC) from PCR-confirmed SARS-CoV-2-positive samples were thawed from -80°C, and used in plaque assays. African green monkey kidney (VeroE6) cells expressing human angiotensin-converting enzyme 2 (ACE2) and transmembrane protease serine 2 precursor (TMPRSS2) were kindly provided by MRC-University of Glasgow Centre for Virus Research (CVR), Glasgow[(1)]. The cells were maintained in Dulbecco's modified Eagle's medium (DMEM; Gibco), 10% fetal calf serum (FCS; Gibco), 1 mg/mL Geneticin (Gibco), 0.2 mg/mL Hygromycin B (Invitrogen).

VeroE6 cell monolayers grown in 12-well plates were washed with Phosphate-buffered saline (PBS) and infected with a 10-fold dilution series of 200µl VTM samples. Plates were incubated at 37°C in 5% CO2 for 1 hour. At 1 hour post-infection, the inoculum was removed and cell monolayers were overlaid with DMEM supplemented with 0.2% w/v bovine serum albumin (Gibco), 0.16% w/v NaHCO3 (Gibco), 10 mM HEPES (Invitrogen), 2 mM L-Gutamine (Gibco), 1X P/S and 0.6% Avicel (Gibco). Following the 72 hours incubation period, the methylcellulose overlay was removed and cells were fixed with 4% paraformaldehyde at room temperature for 20 minutes. The paraformaldehyde was removed, and wells were stained for 1 hour with 1ml of 0.05% crystal violet in 20% methanol. Plates were washed with tap water then dried. Plaques were counted at the dilution in which there were 5-50 plaque forming units (PFUs). The limit of detection of the assay was less than 10 plaque forming units.

Lateral Flow Devices

Lateral flow devices (LFDs) were performed on PCR-positive samples and PCR-negative samples one day before the first and one day after the last PCR-positive sample where available. VTM frozen at -80°C was thawed on ice. Six drops of extract buffer provided with the Innova lateral flow device (LFD) kit were pipetted into the kit extraction tube and homogenised with 100µl of VTM thawed from -80°C. Two drops of this extraction VTM solution were pipetted onto the sample well of the test cartridge and results were read within 30 minutes in

accordance with the manufacturer's instructions. LFDs were graded qualitatively according to the appearance of the T band in comparison to the letter C (control) band and results from the LFDs were photographed and stored as an unmodified file **(Fig.S1)**.

Viral whole genome sequencing for lineage assignments

For WGS performed for pre-alpha, alpha and delta variants, automated RNA extraction was performed using a CyBio FeliX (Analytik Jena) and the innuPREP Virus TS RNA Kit 2.0 (Analytik Jena) according to the manufacturer's instructions, with a sample volume of 200μl, without carrier RNA and with an elution volume of 50μl. RT-qPCR was repeated using an in-house protocol(2). cDNA synthesis was then performed using the LunaScript RT SuperMix Kit (NEB) according to the manufacturer's instructions with a total reaction volume of 20 μl and extracted sample volume of 5μl. Libraries were generated using the EasySeq™ RT-PCR SARS CoV-2 (novel coronavirus) Whole Genome Sequencing kit v2 or v3 (Nimagen) according to the manufacturer's instructions. Samples were then pooled and purified with AMPure XP (Beckman Coulter) magnetic beads. Suitable quality of libraries was confirmed using a Tapestation (Agilent) and concentrations were measured using the Qubit 1x dsDNA High Sensitivity Assay Kit (Thermofisher Scientific) and Qubit 4 Fluorometer (ThermoFisher Scientific). Pooled libraries were then diluted down to 55pM. The final pool was then run on an iSeq 100 (Illumina) with a total of 322 cycles (151 bp paired reads and 10 bp indices). Generated fastq files were processed using the EasySeq variant pipeline (v0.6.0 for kit version 2 or v0.8.1 for kit version 3)(3) which is a Nextflow(4) pipeline that uses fastp(5), BWA MEM(6), SAMtools(7), BCFtools(7), LoFreq(8), mosdepth(9), BEDtools(10), SnpEff(11) and MultiQC(12) to QC, trim and assemble the reads (using reference sequence NC_045512.2) and then generate a consensus sequence and variant report before assigning a PANGO lineage(13) using pangolin (v3.1.17, lineages version 2021-12-06)(14). In the analyses presented here, both genomically-probable and genomically-confirmed cases were included. Pre-alpha status was assigned to cases where alpha infection, and infections caused by other variants of concern or variants under investigation had been excluded.

Modelling viral kinetics

In order to estimate the kinetic parameters of the individual RNA and PFU trajectories, we used a phenomenological Bayesian hierarchical model of the following form

$$v(\tau) = v_{max} \frac{(a+b)}{be^{-a(\tau-\tau_{max})}+ae^{b(\tau-\tau_{max})}},$$

where $v(\tau)$ is the RNA copies/ml or PFU/ml concentration at time τ, v_{max} its maximum value which occurs at time $\tau = \tau_{max}$, a the growth rate and b the decline rate. Exponential growth occurs for times $\tau \ll \tau_{max}$, and exponential decline for times $\tau \gg \tau_{max}$. The model specification allows for test failures with probability p, and for censored data below the detection limit of the assays. The log-likelihood of observing a value x at time τ in the trajectory is given by:

$$l(x) = \log[p\, n(x,\, x_0,\, \sigma_0) + (1-p)n(x,\, \log(v)\,,\, \sigma_v)],$$

where $n(x,\, x_0,\, \sigma_0)$ represents the normal probability density function with mean x_0 and standard deviation σ_0 at point x . False positive and negative test results were assumed to be normally distributed. When the observations are below the limit of detection, $x = 0$, we use the cumulative density function of the normal distribution to account for censored data

$$l(x=0) = \log[p\mathrm{N}(0,\, x_0,\, \sigma_0) + (1-p)\mathrm{N}(0,\, \log(v)\,,\, \sigma_v)],$$

where $\mathrm{N}(x,\, \mu, \sigma)$ is the cumulative density function of the normal distribution with mean μ and standard deviation σ. Since the three parameters a_j, b_j, and $v_{max,\,j}$ for each participant j are positive numbers they were expressed in exponential form: $\theta_{1,j} = \log(a_j)$, $\theta_{2,j} = \log(b_j)$, $\theta_{3,j} = \log(v_{max,\,j})$. The participants were grouped by vaccination status, allowing the kinetic parameters of each group to be represented with a different group mean.

Using a non-centred parametrisation to allow for a more efficient exploration of the parameter space, the parameters of each participant were expressed as

$$\theta_{i,\,j} \;=\; \mu_{i,\,k(j)} \;+\; \delta_i \, z_{i,\,j},$$

where $k = \{1{,}2\}$, with 1 being unvaccinated, and 2 being vaccinated participants, and each participant j mapped to one of the two groups via $k(j)$. In the sensitivity analysis based on a smaller cohort consisting of participants with at least 5 positive PFU samples, we fitted a single group ($k = 1$) in order to compare the mean viral RNA and PFU kinetic parameters. Deviations from the mean were modelled using a multiplier δ_i times $z_{i,\,j}$, with prior

$$z_i \;\sim\; n(0,\, C),$$

where z_i is a three-dimensional vector with elements $z_i \;=\; \{z_{1,\,i}\,,\, z_{2,\,i}\,,\, z_{3,\,i}\}$, $n(0,\, C)$ a three-dimensional multivariate normal distribution, and C a correlation matrix with prior $lkj\_corr\_cholesky(1)$, using the Lewandowski-Kurowicka-Joe (LKJ) prior with Cholesky factor representation. Similar to Singanayagam *et al*, we used weakly informative priors and performed prior predictive checks to ensure that the selected priors were consistent with previously published viral trajectories[(15)]. $\mu_1 \;\sim\; n(15,\, 15)$ (for RNA trajectories), $\mu_1 \;\sim\; n(12,\, 12)$ (for PFU trajectories)$\mu_2 \;\sim\; n(1.25,\, 0.75)$, $\mu_3 \;\sim\; n(0.5,\, 1.4)$, $\delta_1 \;\sim\; n_{>0}\,(0,\, 10)$, $\delta_2 \;\sim\; n_{>0}\,(0,\, 1)$, $\delta_3 \;\sim\; n_{>0}\,(0,\, 1)$, with $n_{>0}$ indicating normal distributions truncated at zero from below. The priors were chosen to allow for a broad range of values of the kinetic parameters and were compatible with the trajectories of previously investigated infecting variants.

Time τ was measured in days and defined on a participant-specific basis such that $\tau \;=\; 0$ was the first study day, and the time of peak was given the prior probability $\tau_{max} \sim n(5{,}5)$.

The error probability p was fitted on a log scale such that $(-\log\, p\;) \;\sim\; n_{\geq 0}(5{,}2)$ (i.e. giving a relatively uninformative truncated [above at 1] lognormal prior for p with mean 0.04 and standard deviation 0.12) while x_0 was given a prior of $n(0{,}1)$ (giving a 1:1 ratio of false positives and negatives). Both σ_0 and σ_v were given the relatively uninformative prior of $n_{\geq 1}(3{,}3)$, where the minimum lower bound of 1 prevented Markov Chain Monte Carlo (MCMC) divergence issues associated with exploring very low values of these measurement precision related parameters. In order to avoid artificially steep gradients when the viral load crossed the assay limit of detection (LoD) thresholds, the LoD was subtracted from all data points in the modelling (such that the lowest data points would be at zero) and was afterwards added to the estimated viral trajectories. The limits of detection were given as 3.4 and 2.3 in natural logarithm units for the RT-PCR and plaque assays, respectively.

Using the individually fitted trajectories we calculated the within-sample means of the kinetic parameters as

$$\log\!\left(v_{max,\,k}\right) \;=\; \Sigma_{j\,\in\,\{j\,|\,k(j)\,=\,k\}}\theta_{1,\,j}\,/\,n_k\;,$$

$$a_k \;=\; \Sigma_{j\,\in\{j\,|\,k(j)=k\}}\exp\!\left(\theta_{2,\,j}\right)\;/\,n_k\;,$$

$$b_k \;=\; \Sigma_{j\,\in\{j\,|\,k(j)=k\}}\exp\!\left(\theta_{3,\,j}\right)\;/\,n_k\;.$$

Posterior probabilities that the group mean estimates of the viral RNA and PFU kinetic parameters were different were calculated based on the MCMC samples μ_i by

$$p \;=\; \sum_i^{N_S}\delta(\mu_i^{PFU} > \mu_i^{RNA})\;/\,N_S\,,$$

where $\delta(x > y)$ is 1 if $x > y$ and 0 otherwise, and N_S is the number of samples.

Modelling was conducted in R version 4.1.2[(16)] using the package RStan[(17)] to fit the models using Hamiltonian MCMC methods. For each model fit, 8 MCMC chains of 8,000 iterations each were undertaken. The first 3,000 iterations of each chain were used during the burn-in phase, and each chain was thinned by half, giving a total of 20,000 posterior samples for each model. Standard Stan diagnostics were used to confirm convergence, mixing and adequate effective sample sizes.

Supplementary Methods References

1. Rihn SJ, Merits A, Bakshi S, Turnbull ML, Wickenhagen A, Alexander AJT, et al. A plasmid DNA-launched SARS-CoV-2 reverse genetics system and coronavirus toolkit for COVID-19 research. PLOS Biology [Internet]. 2021 Feb 25;19(2):e3001091-. Available from: https://doi.org/10.1371/journal.pbio.3001091

2. Rowan AG, May P, Badhan A, Herrera C, Watber P, Penn R, et al. Optimized protocol for a quantitative SARS-CoV-2 duplex RT-qPCR assay with internal human sample sufficiency control. Journal of Virological Methods [Internet]. 2021;294:114174. Available from: https://www.sciencedirect.com/science/article/pii/S0166093421001130

3. Wolters F, Coolen JPM, Tostmann A, van Groningen LFJ, Bleeker-Rovers CP, Tan ECTH, et al. Novel SARS-CoV-2 Whole-genome sequencing technique using Reverse Complement PCR enables easy, fast and accurate outbreak analysis in hospital and community settings. bioRxiv [Internet]. 2020 Jan 1;2020.10.29.360578. Available from: http://biorxiv.org/content/early/2020/11/25/2020.10.29.360578.abstract

4. di Tommaso P, Chatzou M, Floden EW, Barja PP, Palumbo E, Notredame C. Nextflow enables reproducible computational workflows. Nature Biotechnology [Internet]. 2017;35(4):316–9. Available from: https://doi.org/10.1038/nbt.3820

5. Chen S, Zhou Y, Chen Y, Gu J. fastp: an ultra-fast all-in-one FASTQ preprocessor. Bioinformatics [Internet]. 2018 Sep 1;34(17):i884–90. Available from: https://doi.org/10.1093/bioinformatics/bty560

6. Li H. Aligning sequence reads, clone sequences and assembly contigs with BWA-MEM [Internet]. 2013. Available from: http://github.com/lh3/bwa.

7. Danecek P, Bonfield JK, Liddle J, Marshall J, Ohan V, Pollard MO, et al. Twelve years of SAMtools and BCFtools. GigaScience [Internet]. 2021 Feb 1;10(2):giab008. Available from: https://doi.org/10.1093/gigascience/giab008

8. Wilm A, Aw PPK, Bertrand D, Yeo GHT, Ong SH, Wong CH, et al. LoFreq: a sequence-quality aware, ultra-sensitive variant caller for uncovering cell-population heterogeneity from high-throughput sequencing datasets. Nucleic Acids Research [Internet]. 2012 Dec 1;40(22):11189–201. Available from: https://doi.org/10.1093/nar/gks918

9. Pedersen BS, Quinlan AR. Mosdepth: quick coverage calculation for genomes and exomes. Bioinformatics [Internet]. 2018 Mar 1;34(5):867–8. Available from: https://doi.org/10.1093/bioinformatics/btx699

10. Quinlan AR, Hall IM. BEDTools: a flexible suite of utilities for comparing genomic features. Bioinformatics [Internet]. 2010 Mar 15;26(6):841–2. Available from: https://doi.org/10.1093/bioinformatics/btq033

11. Cingolani P, Platts A, Wang LL, Coon M, Nguyen T, Wang L, et al. A program for annotating and predicting the effects of single nucleotide polymorphisms, SnpEff. Fly [Internet]. 2012 Apr 1;6(2):80–92. Available from: https://doi.org/10.4161/fly.19695

12. Ewels P, Magnusson M, Lundin S, Käller M. MultiQC: summarize analysis results for multiple tools and samples in a single report. Bioinformatics [Internet]. 2016 Oct 1;32(19):3047–8. Available from: https://doi.org/10.1093/bioinformatics/btw354

13. Rambaut A, Holmes EC, O'Toole Á, Hill V, McCrone JT, Ruis C, et al. A dynamic nomenclature proposal for SARS-CoV-2 lineages to assist genomic epidemiology. Nature Microbiology [Internet]. 2020;5(11):1403–7. Available from: https://doi.org/10.1038/s41564-020-0770-5

14. O'Toole Á, Scher E, Underwood A, Jackson B, Hill V, McCrone JT, et al. Assignment of epidemiological lineages in an emerging pandemic using the pangolin tool. Virus Evolution [Internet]. 2022 Jan 15;7(2):veab064. Available from: https://doi.org/10.1093/ve/veab064

15. Singanayagam A, Hakki S, Dunning J, Madon KJ, Crone MA, Koycheva A, et al. Community transmission and viral load kinetics of the SARS-CoV-2 delta (B.1.617.2) variant in vaccinated and unvaccinated individuals in the UK: a prospective, longitudinal, cohort study. The Lancet Infectious Diseases [Internet]. 2022 Feb 1;22(2):183–95. Available from: https://doi.org/10.1016/S1473-3099(21)00648-4

16. R Core Team. R: A language and environment for statistical computing [Internet]. R Foundation for Statistical Computing. 2021 [cited 2022 Feb 22]. Available from: https://www.R-project.org

17. Stan Development Team. {RStan}: the {R} interface to {Stan} [Internet]. R package version 2.21.3. 2021 [cited 2022 Feb 22]. Available from: https://mc-stan.org
